# Supplementary material for: The Influence of Phytosociological Cultivation and Fertilization on Polyphenolic Content of Menthae and Melissae folium and Evaluation of Antioxidant Properties through In Vitro and In Silico Methods
Source: Plants (Basel). 2022 Sep 14;11(18):2398. doi: 10.3390/plants11182398 (PMC9504945; doi:10.3390/plants11182398)
Supplement: Supplementary file 1 [file plants-11-02398-s001.zip › plants-1907022-supplementary.pdf]

S1. Result

S1.1. FT-ICR MS chromatograms

ESI+

Mentha piperita L.

Green colour chromatogram – MM E (mint extract from control crop)

Violet colour chromatogram – MF E (mint extract from common crop)

Grey colour chromatogram – theoretical peak obtained by the computer program

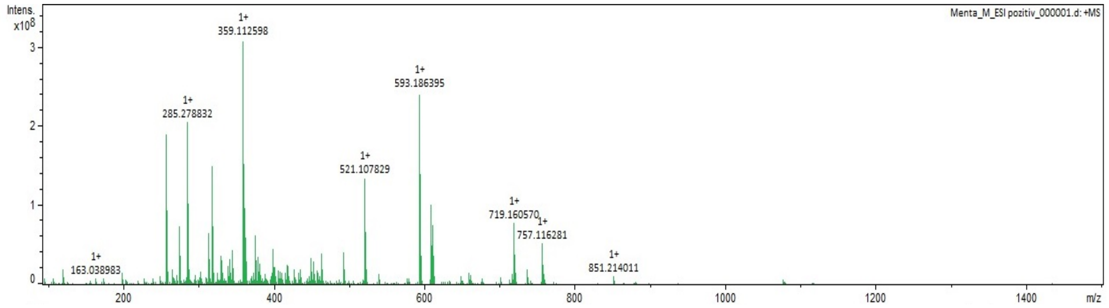

a. MM E mass spectrum

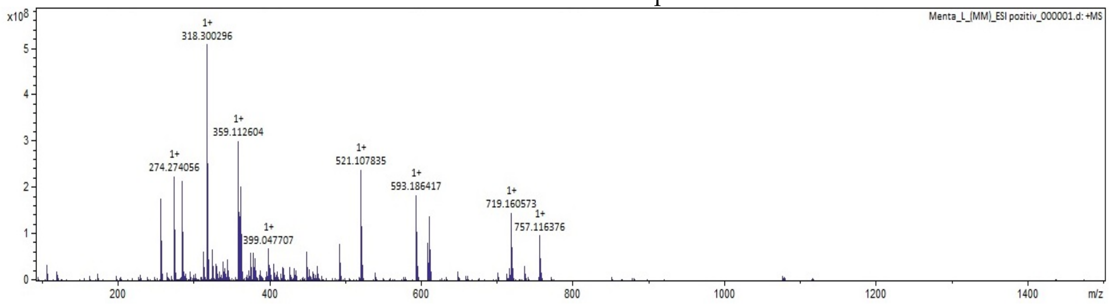

b. MF E mass spectrum

Figure S1. (a) and (b) – entire mass spectra obtained on positive ionization.

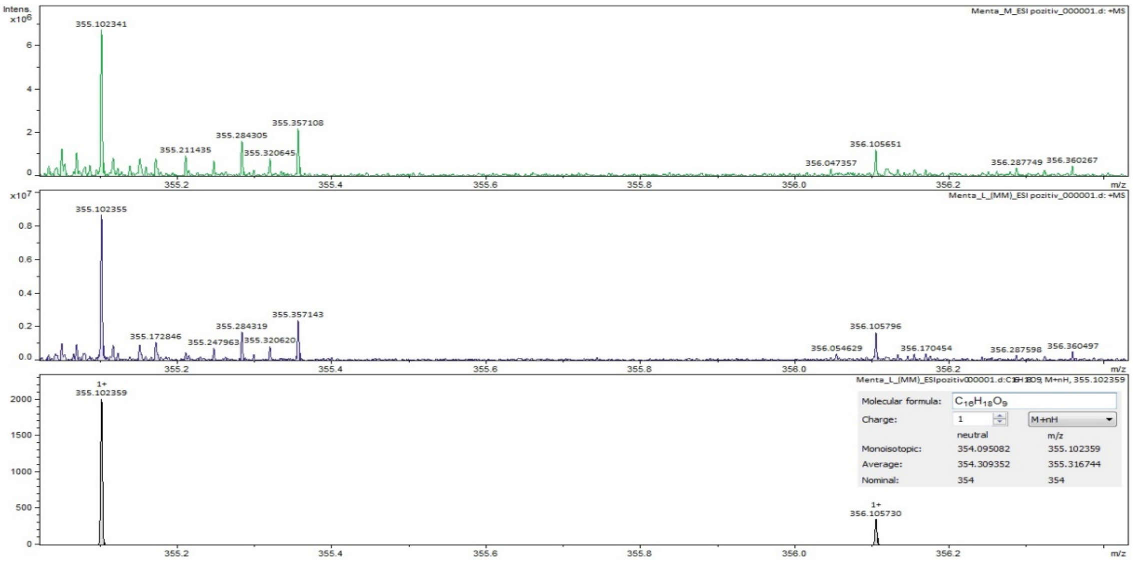

Figure S2. Chlorogenic acid ($C_{16}H_{18}O_9$) – m/z is 355.10, ESI+.

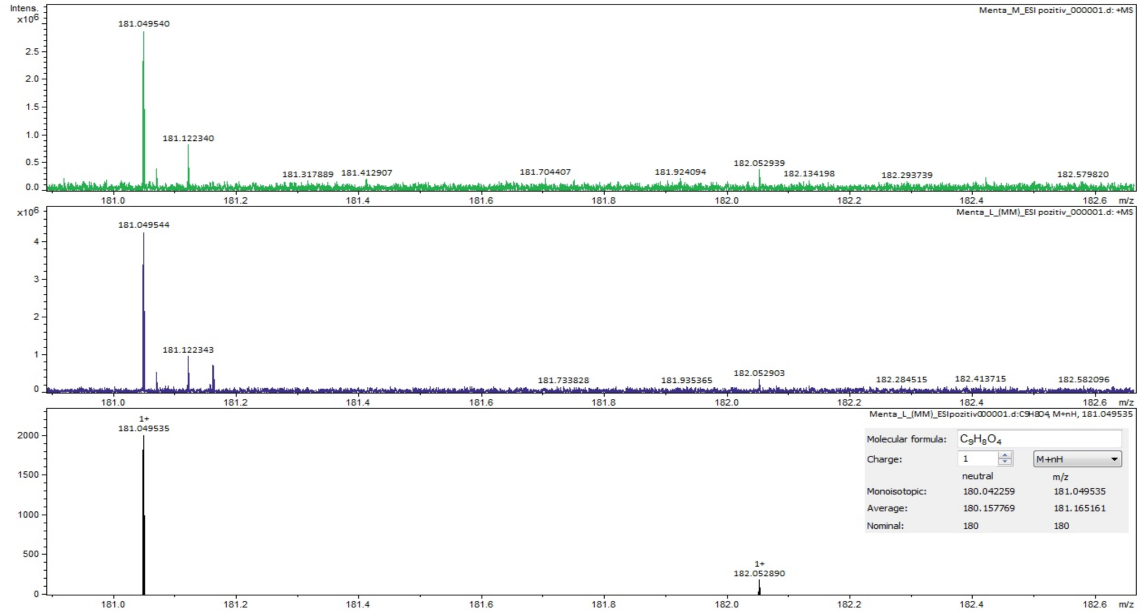

a

b

c

Figure S3. Caffeic acid ($C_9H_8O_4$) – m/z is 181.05, ESI+.

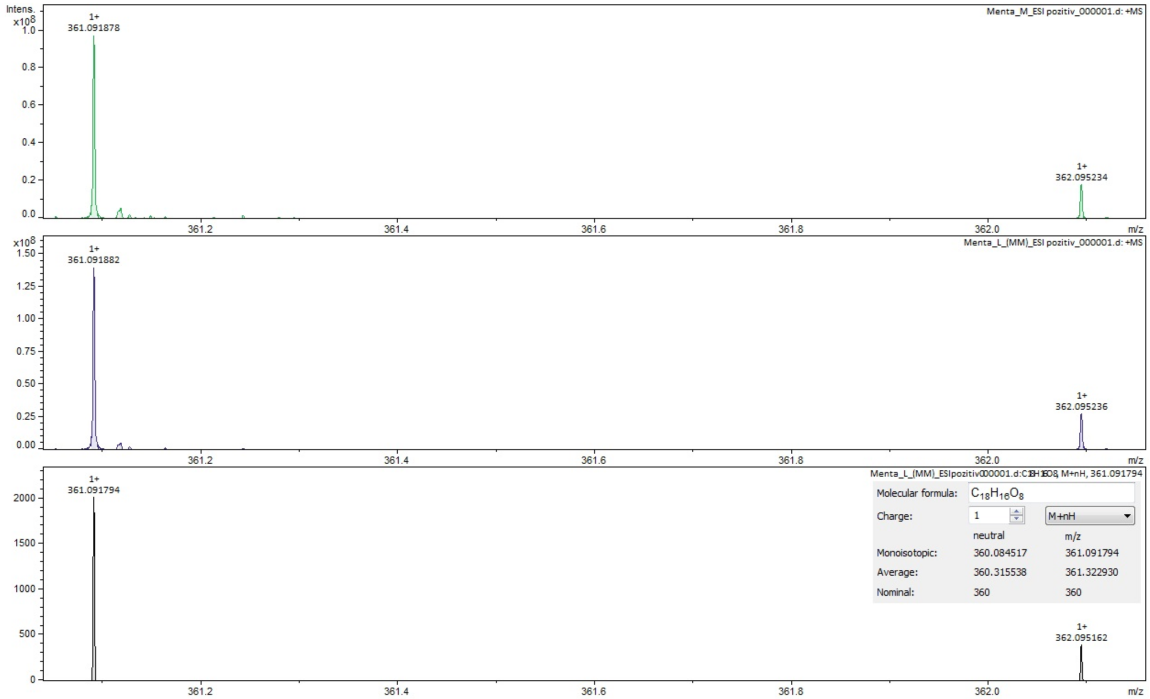

Figure S4. Rosmarinic acid ($C_{18}H_{16}O_8$) – m/z is 361.09, ESI+.

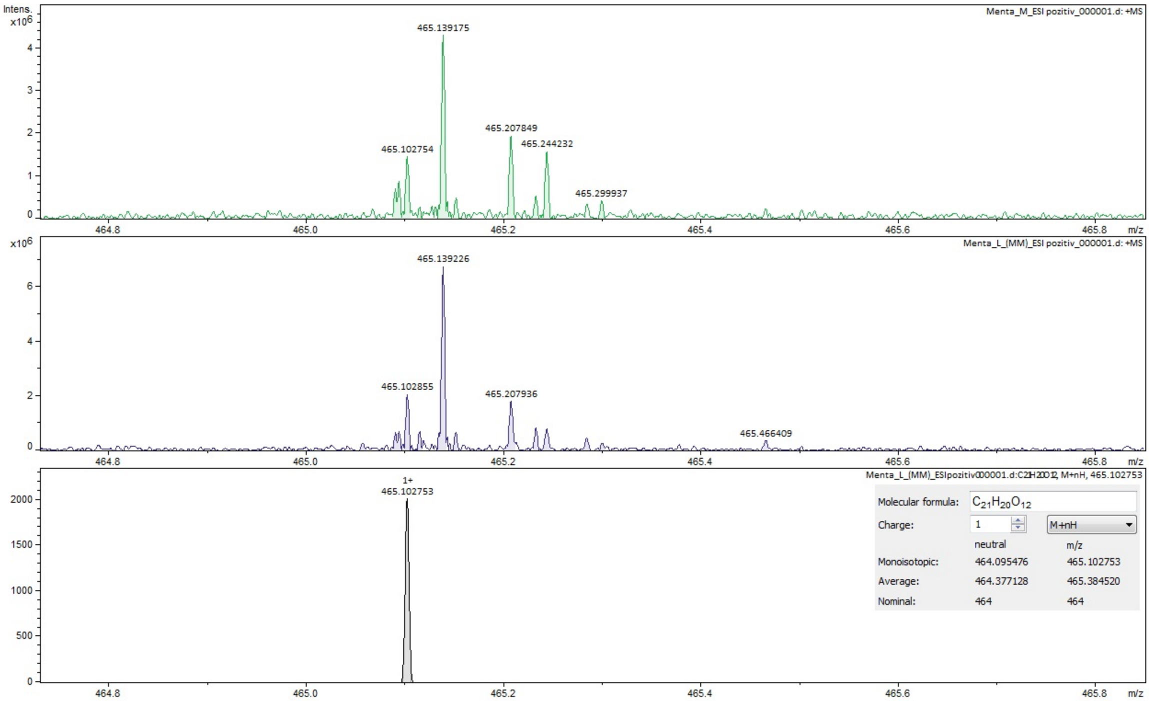

Figure S5. Isoquercitrin ($C_{21}H_{20}O_{12}$) – m/z is 465.10, ESI+.

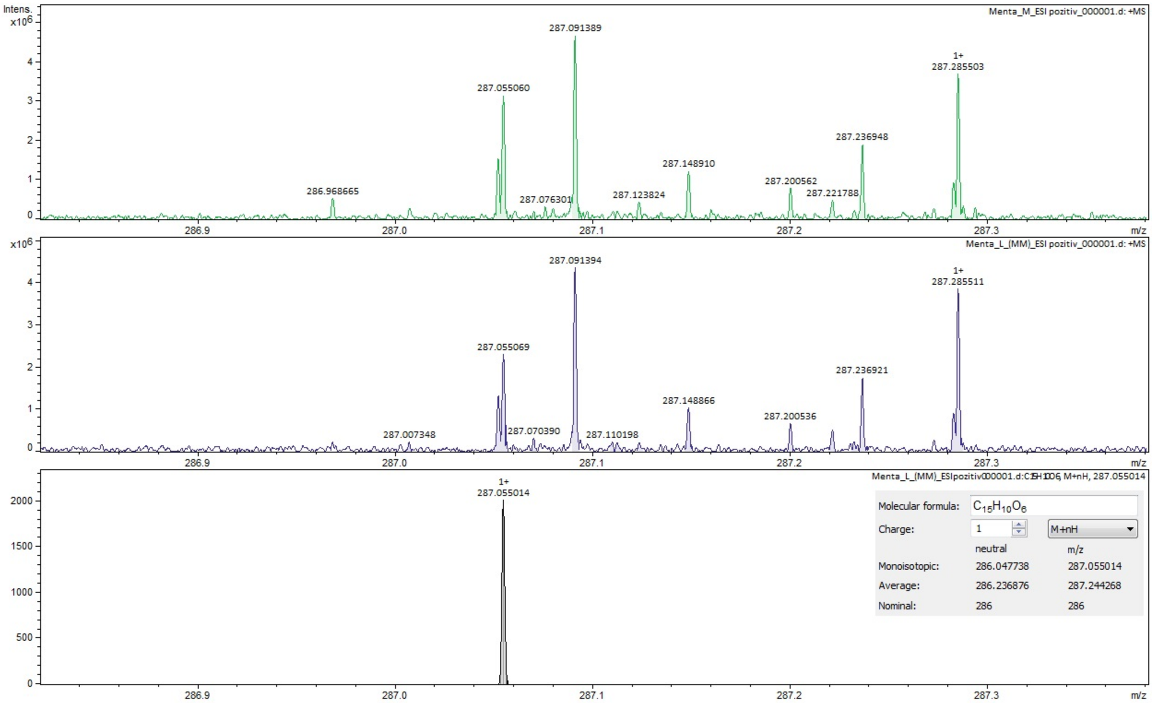

Figure S6. Luteolin + Kaempferol ($C_{15}H_{10}O_6$) – m/z is 287.06, ESI+.

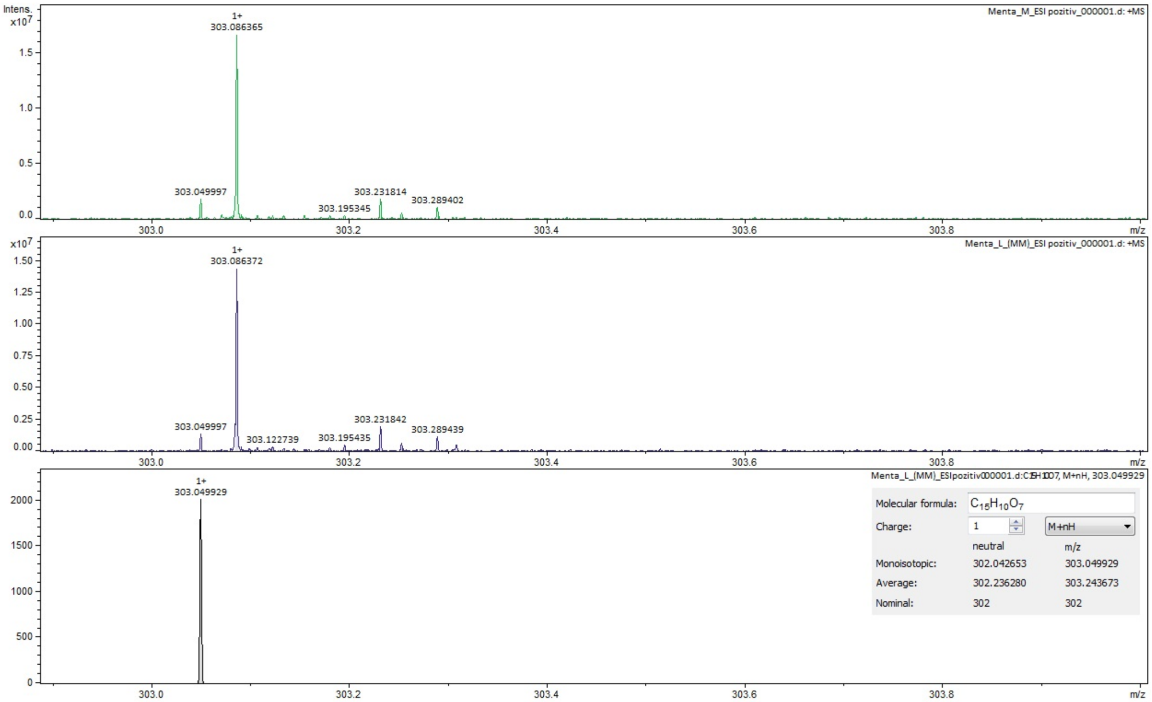

Figure S7. Quercetin ($C_{15}H_{10}O_7$) – m/z is 303.05, ESI+.

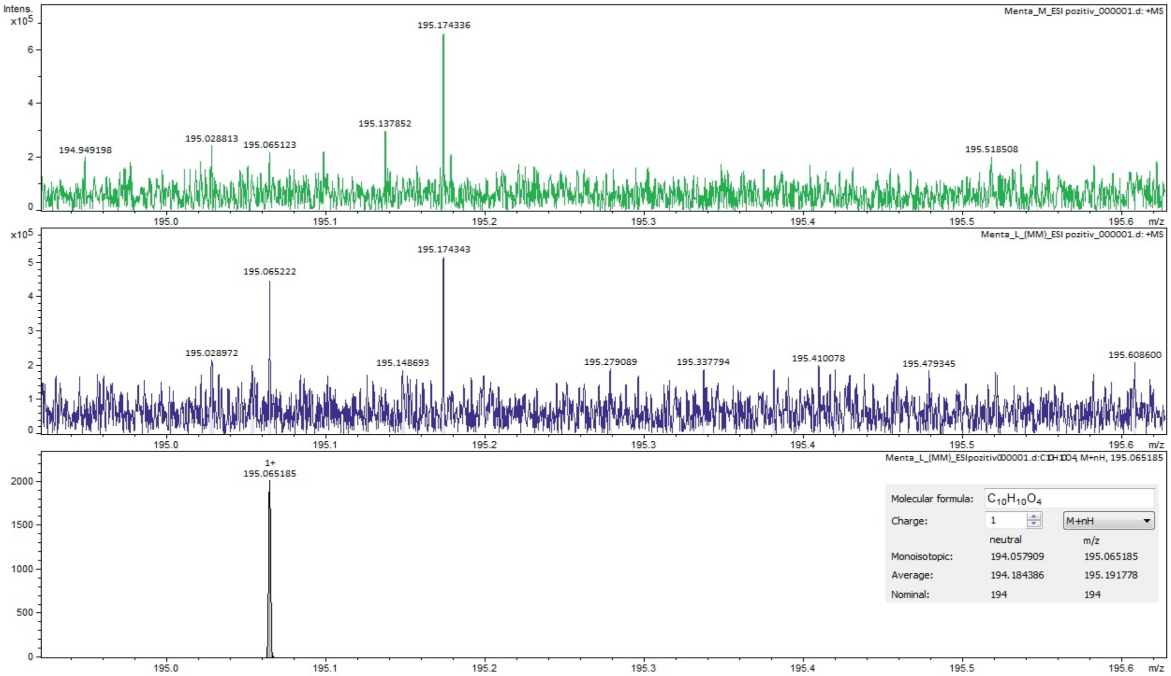

Figure S8. Ferulic acid ($C_{15}H_{10}O_6$) – m/z is 195.07, ESI+.

Melissa officinalis L.
Green colour chromatogram – MLM E (lemon balm extract from control crop)
Brown colour chromatogram – MLF E (lemon balm extract from common crop)
Grey colour chromatogram – theoretical peak obtained by the computer program

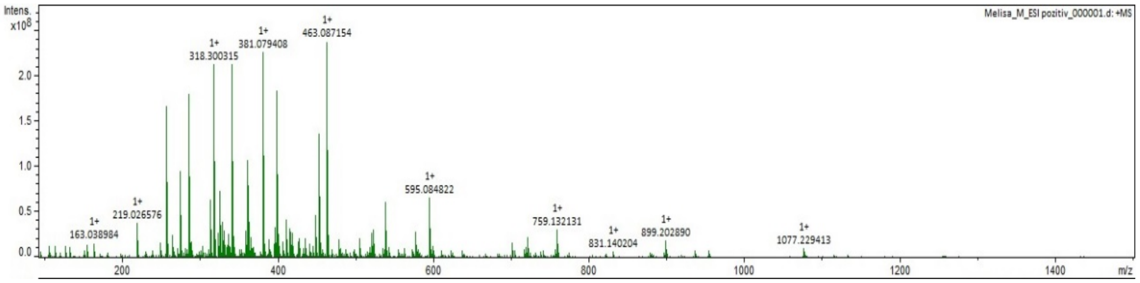

a. MLM E mass spectrum

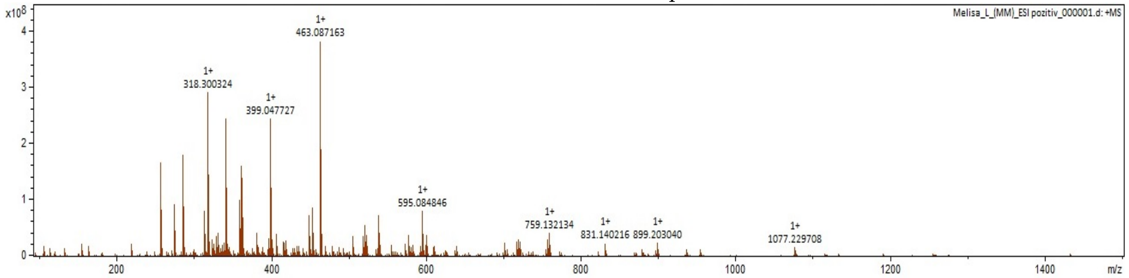

b. MLF E mass spectrum

Figure S9. (a) and (b) – entire mass spectra obtained on positive ionization.

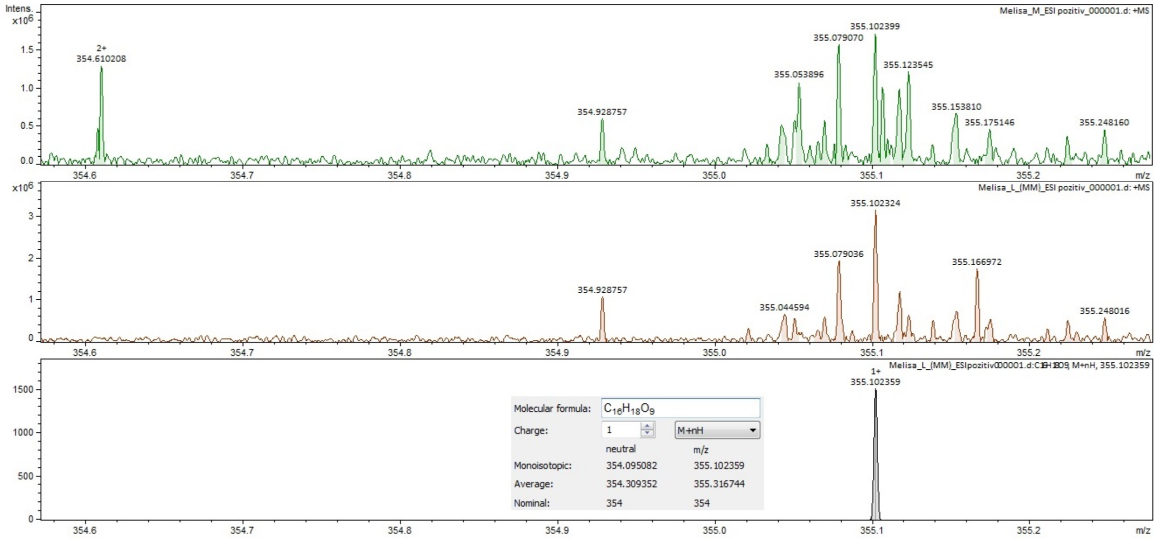

Figure S10. Chlorogenic acid ($C_{16}H_{18}O_9$) – m/z is 355.10, ESI+.

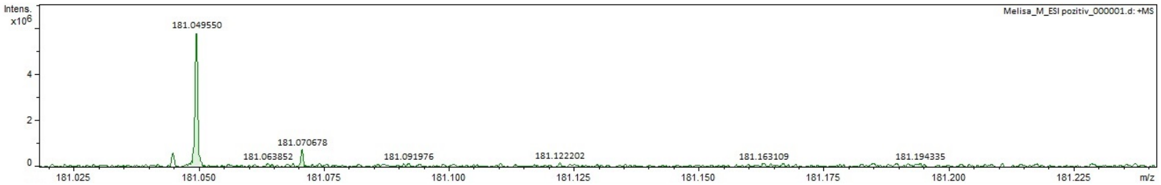

a

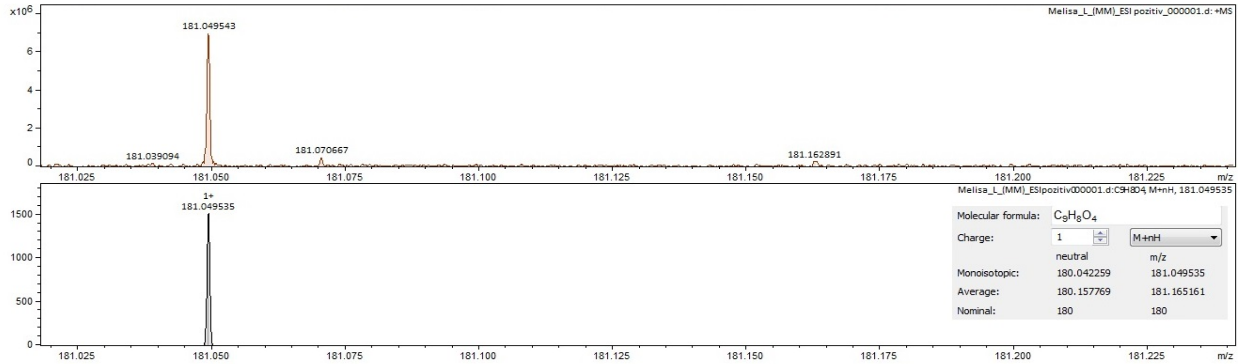

b

c

Figure S11. Caffeic acid ($C_9H_8O_4$) – m/z is 181.05, ESI+.

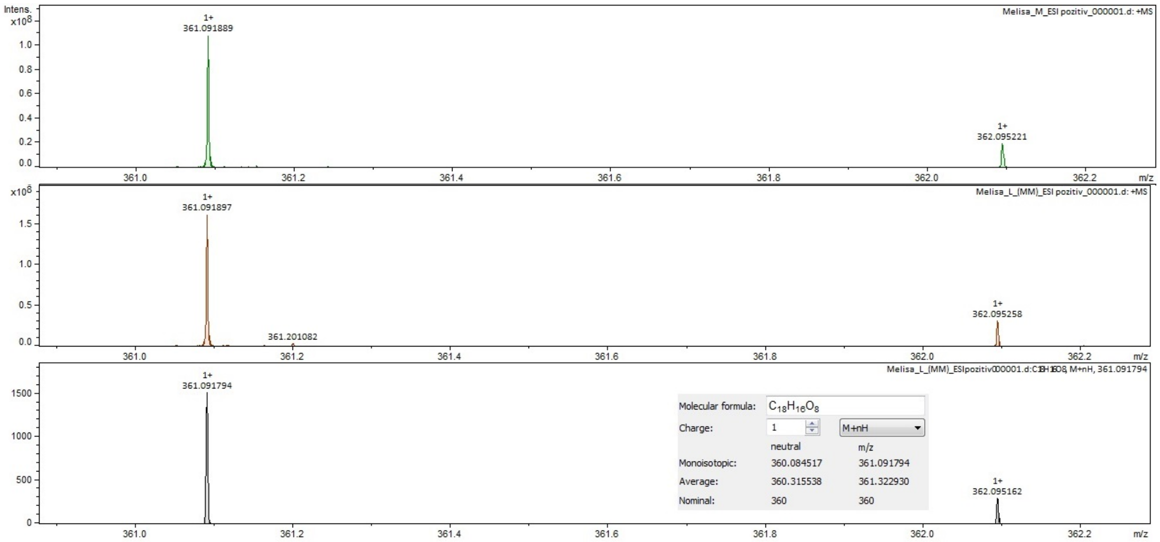

Figure S12. Rosmarinic acid ($C_{18}H_{16}O_8$) – m/z is 361.09, ESI+.

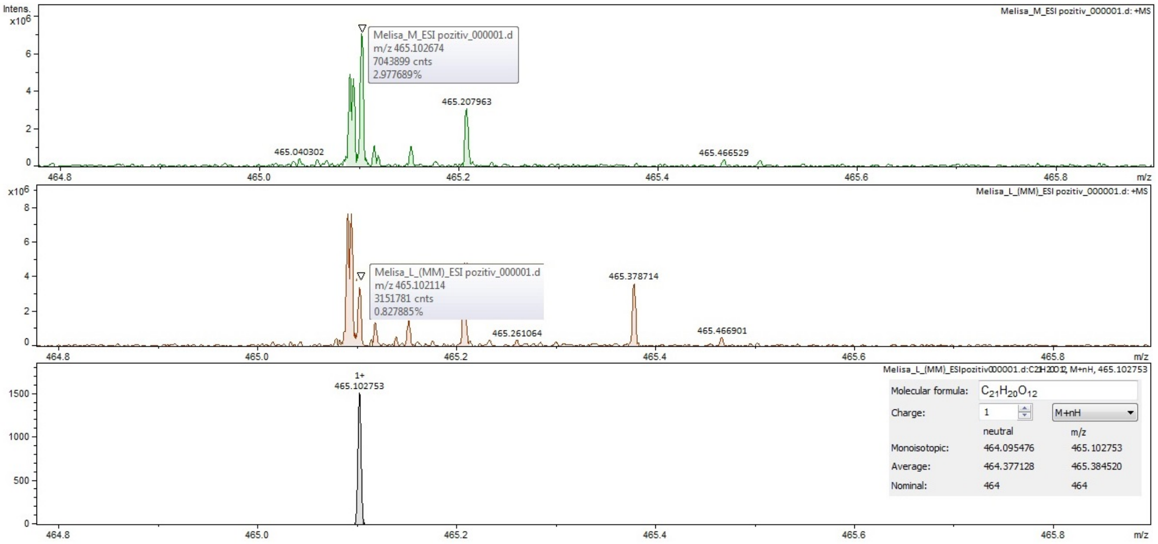

Figure S13. Isoquercitrin ($C_{21}H_{20}O_{12}$) – m/z is 465.10, ESI+.

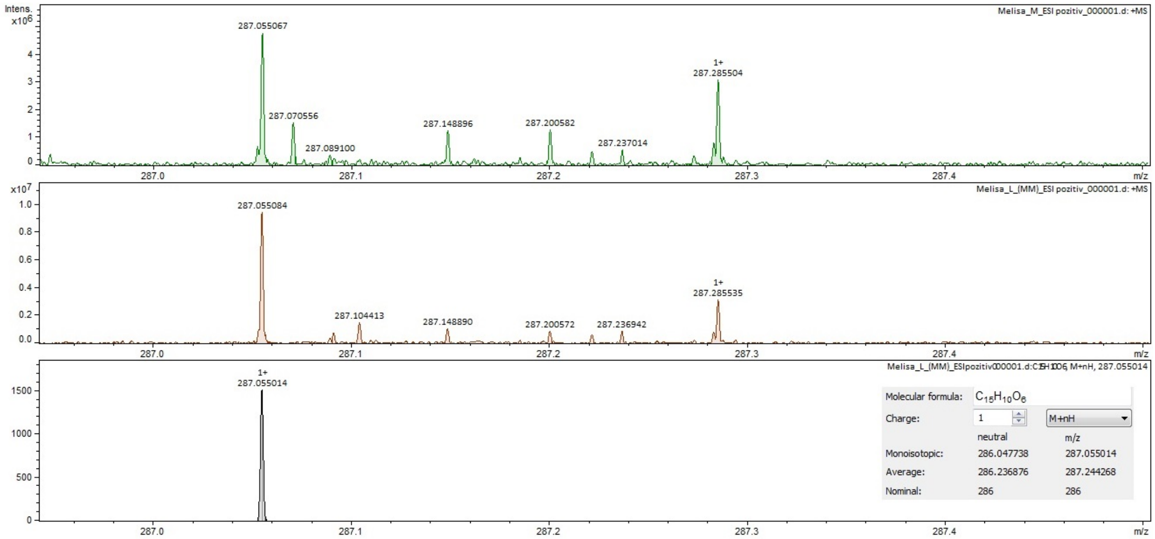

Figure S14. Luteolin + Kaempferol ($C_{15}H_{10}O_6$) – m/z is 287.06, ESI+.

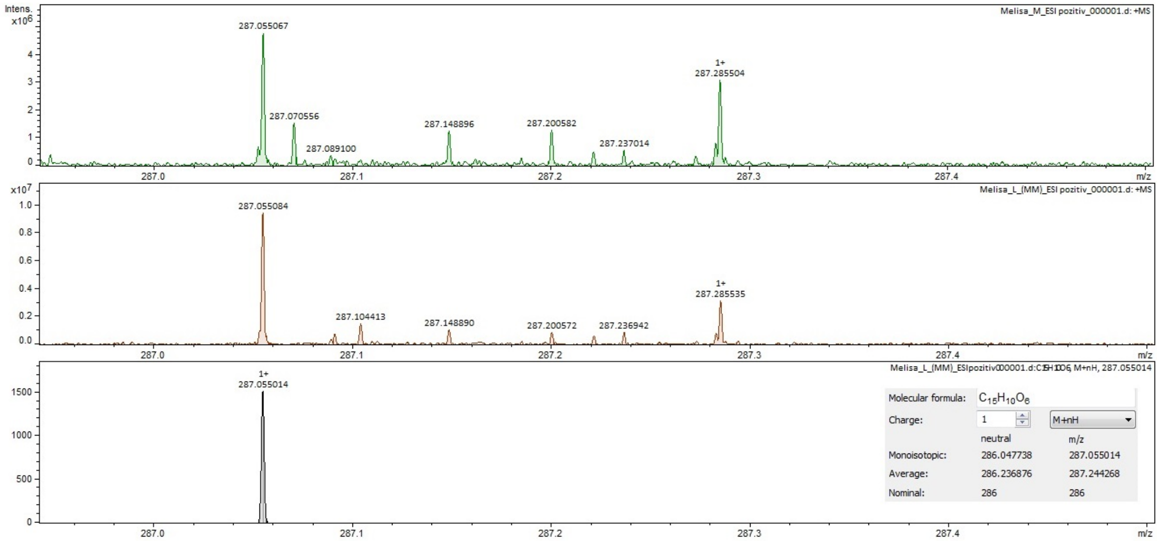

Figure S15. Quercetin ($C_{15}H_{10}O_7$) – m/z is 303.05, ESI+.

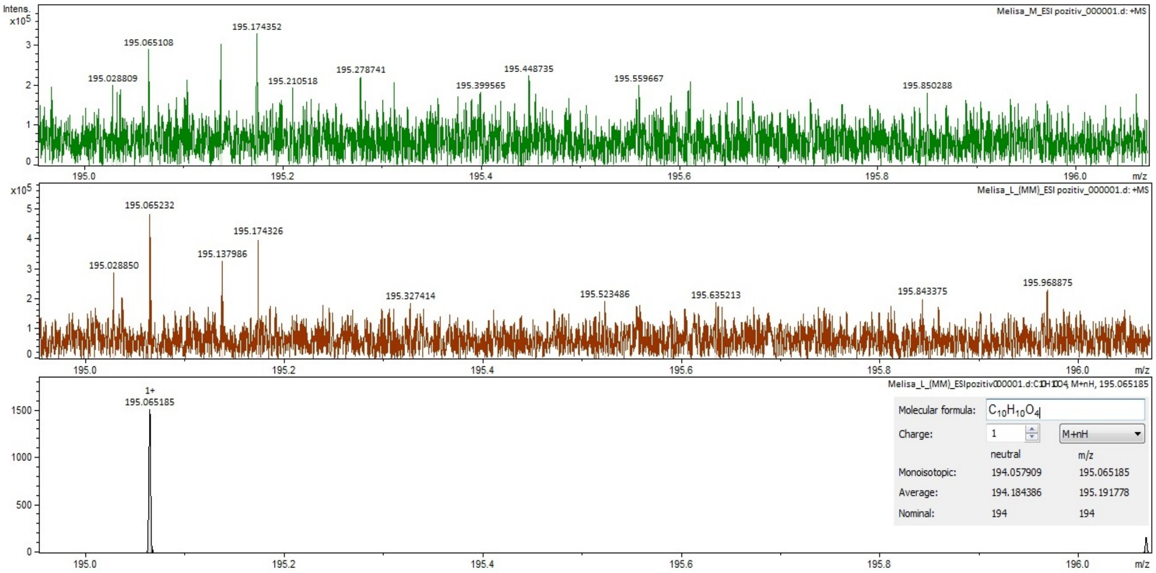

Figure S16. Ferulic acid ($C_{15}H_{10}O_6$) – m/z is 195.07, ESI+.

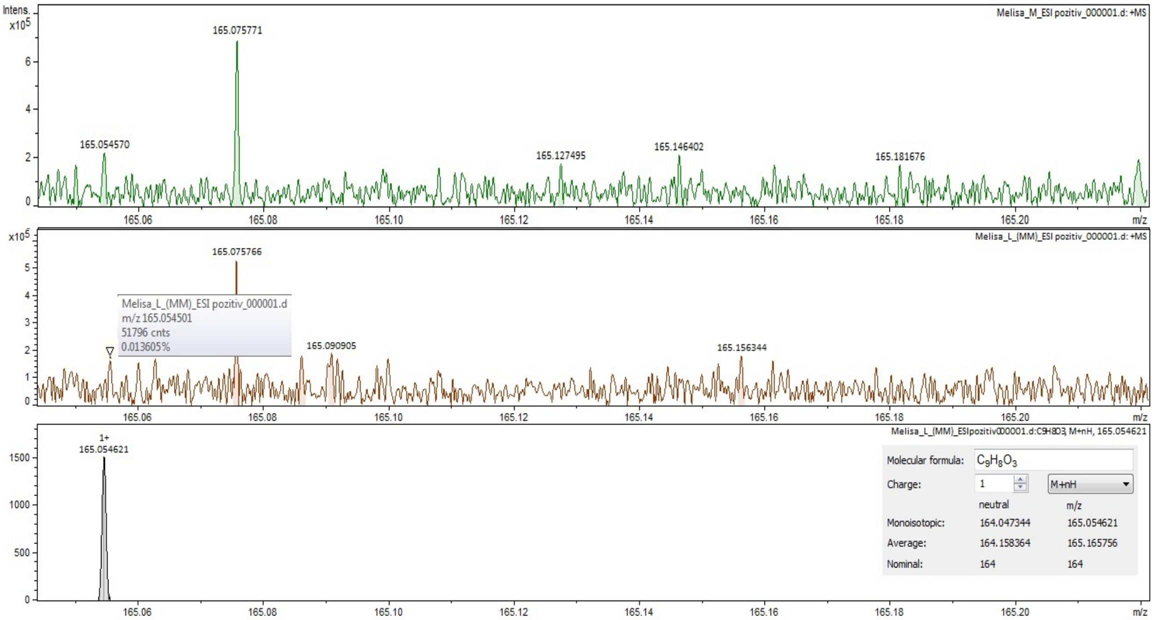

Figure S17. *p*-Coumaric acid ($C_9H_8O_3$) – m/z is 165.05, ESI+.

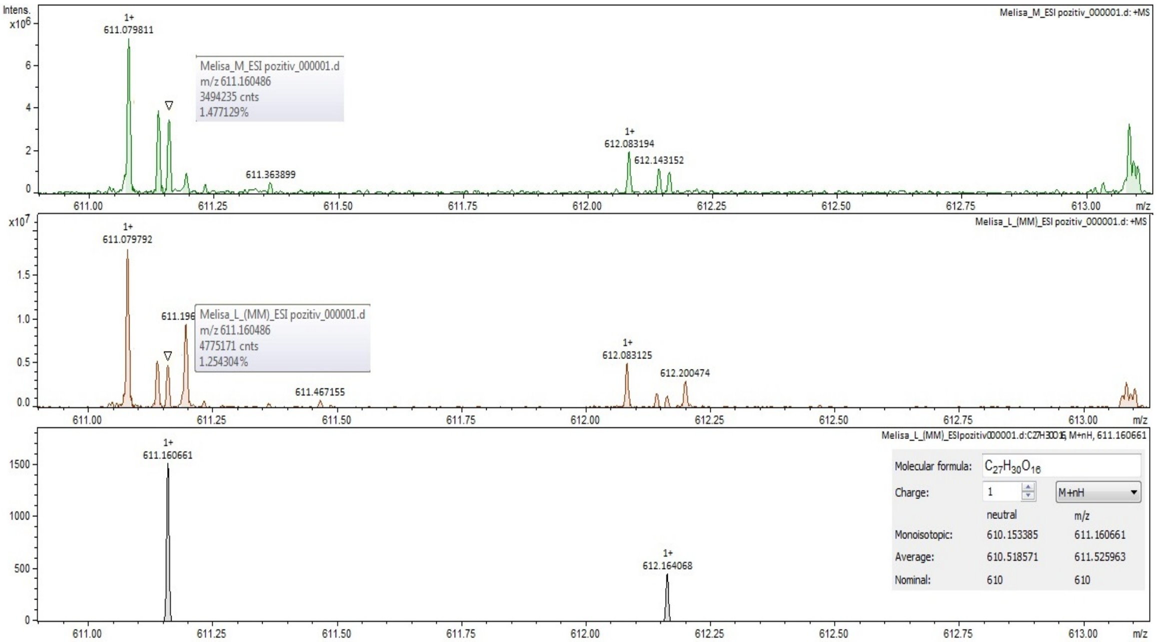

Figure S18. Rutin ($C_{27}H_{30}O_{16}$) – m/z is 611.16, ESI+.

ESI-

Mentha piperita L.

Green colour chromatogram – MM E (mint extract from control crop)

Violet colour chromatogram – MF E (mint extract from common crop)

Grey colour chromatogram – theoretical peak obtained by the computer program

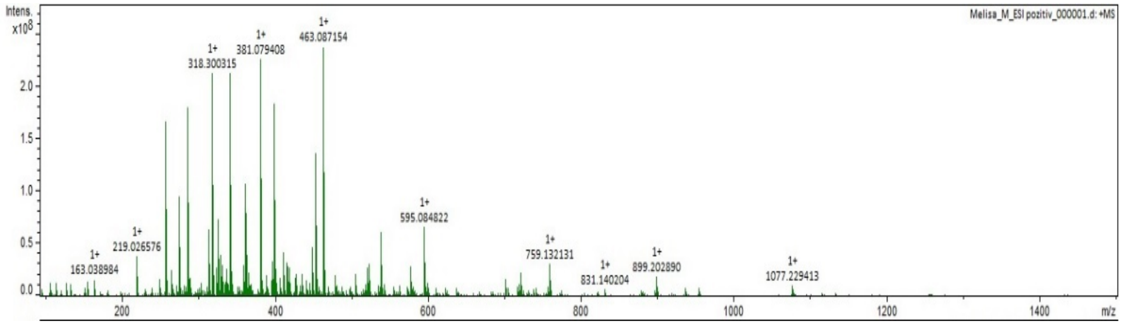

a. MM E mass spectrum

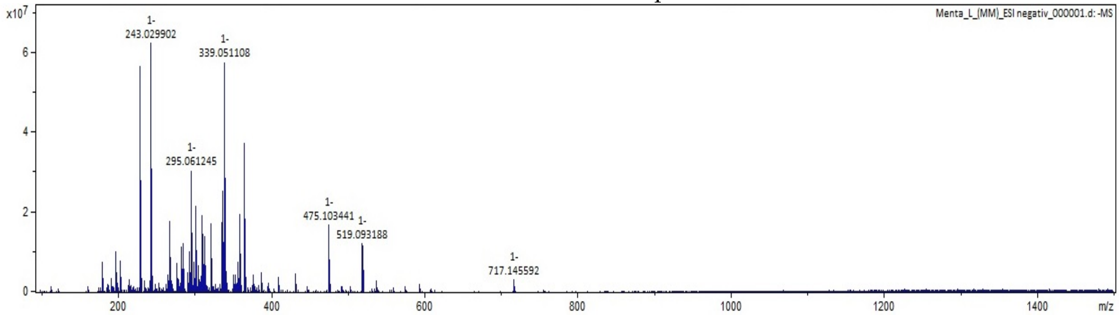

b. MF E mass spectrum

Figure S19. (a) and (b) – entire mass spectra obtained on negative ionization.

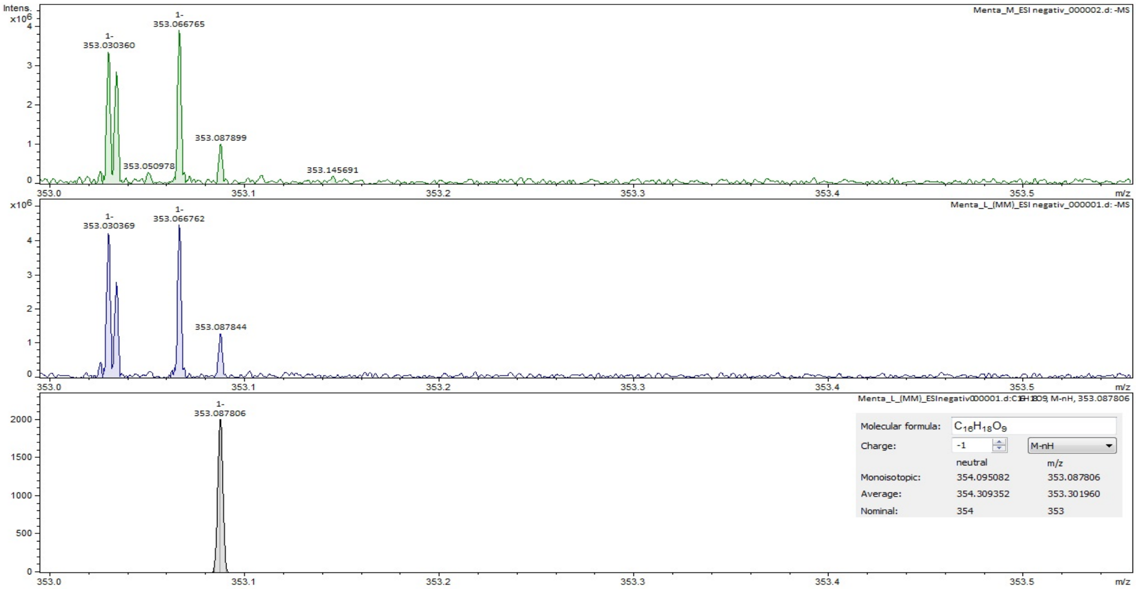

Figure S20. Chlorogenic acid ($C_{16}H_{18}O_9$) – m/z is 353.09, ESI-.

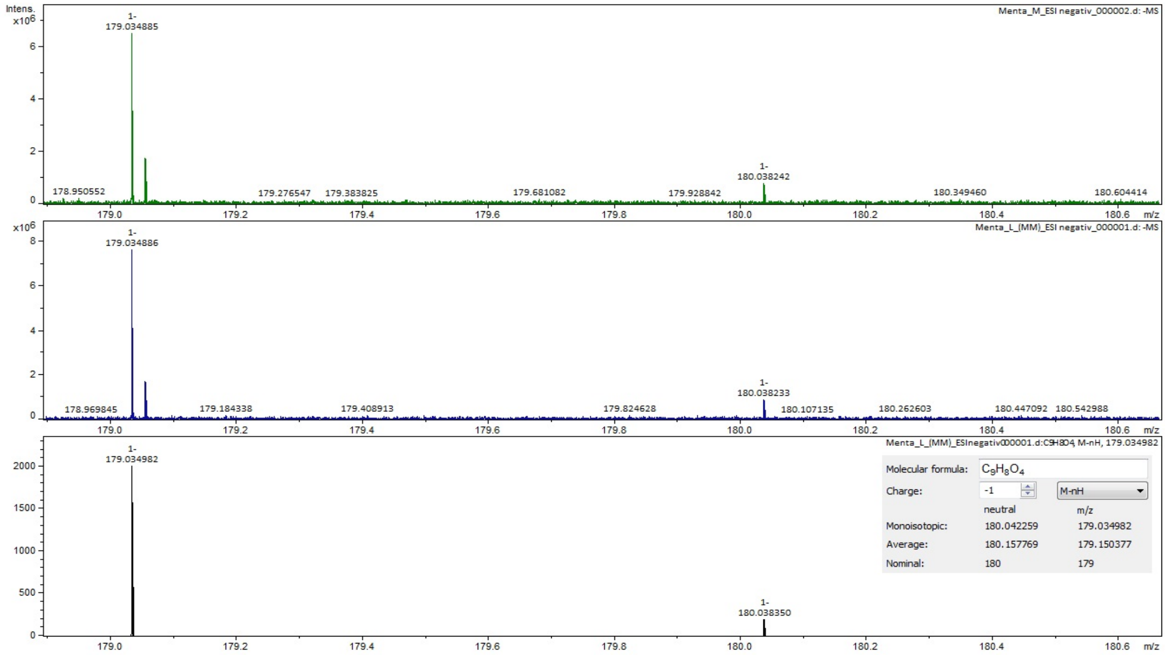

Figure S21. Caffeic acid ($C_9H_8O_4$)– m/z is 179.03, ESI-.

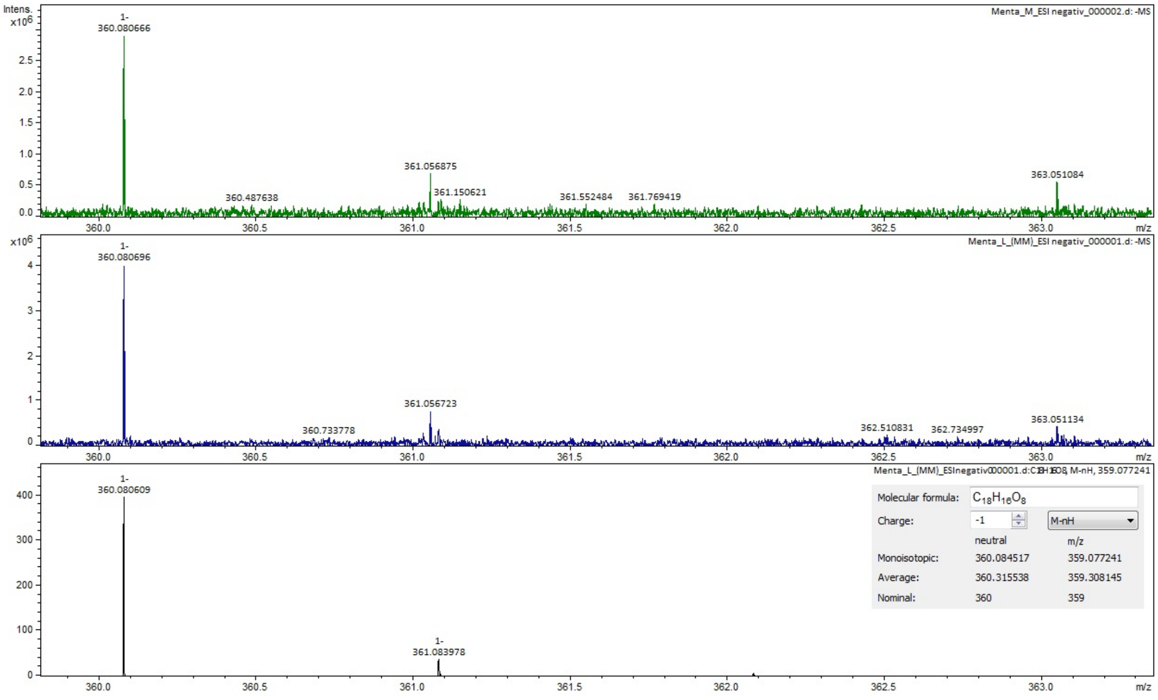

Figure S22. Rosmarinic acid ($C_{18}H_{16}O_8$) – m/z is 359.08, ESI-.

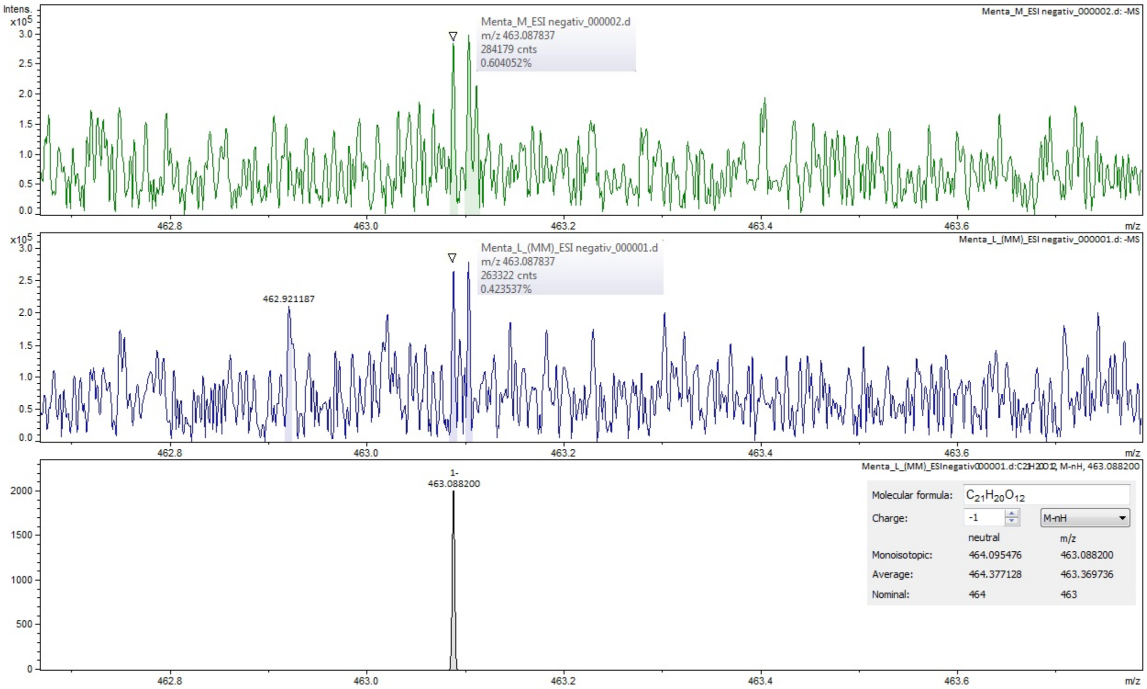

Figure S23. Isoquercitrin ($C_{21}H_{20}O_{12}$) – m/z is 463.09, ESI-.

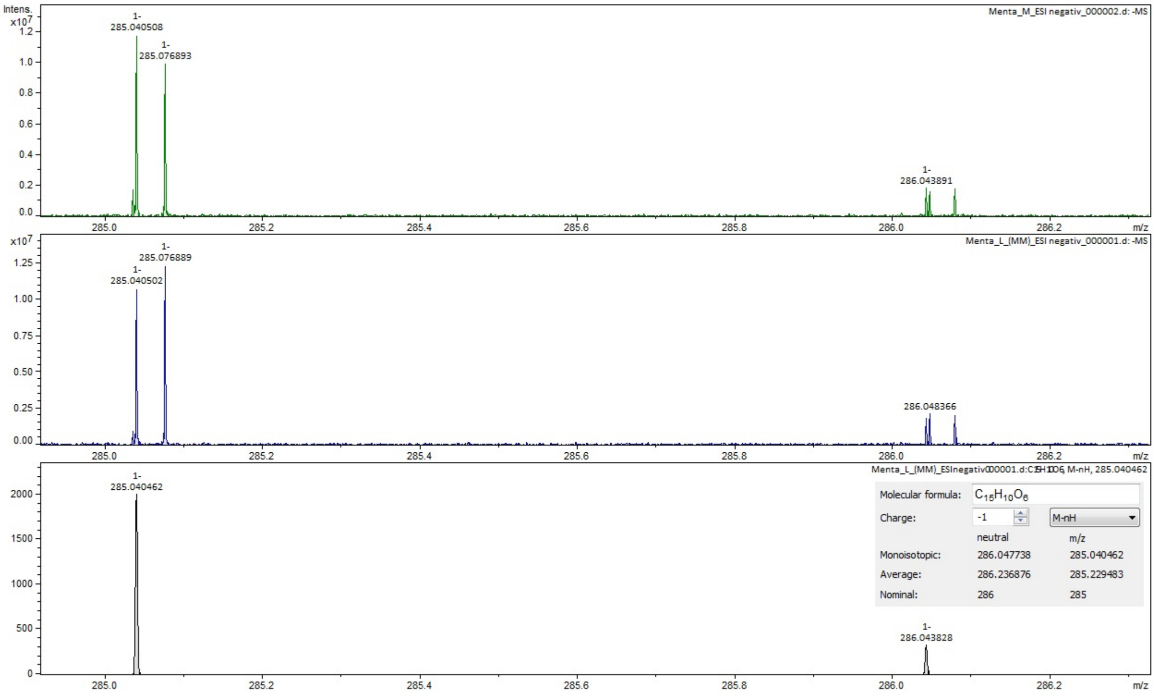

Figure S24. Luteolin + Kaempferol ($C_{15}H_{10}O_{6}$) – m/z is 285.04, ESI-.

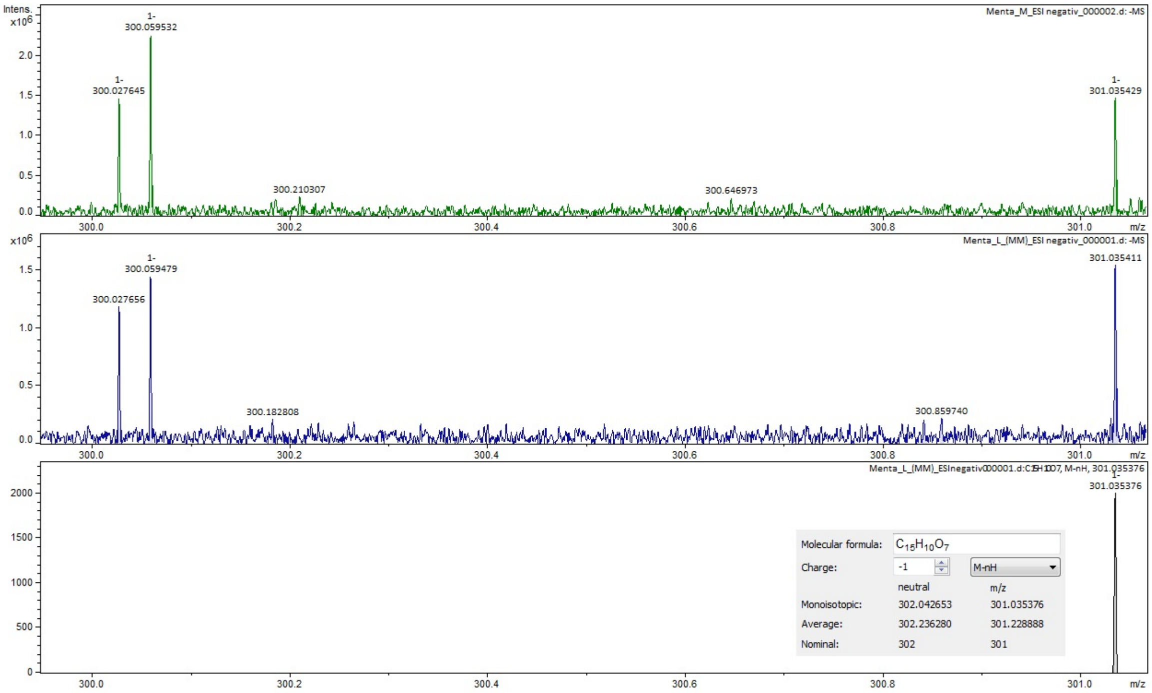

Figure S25. Quercetin ($C_{15}H_{10}O_7$) – m/z is 301.04, ESI-.

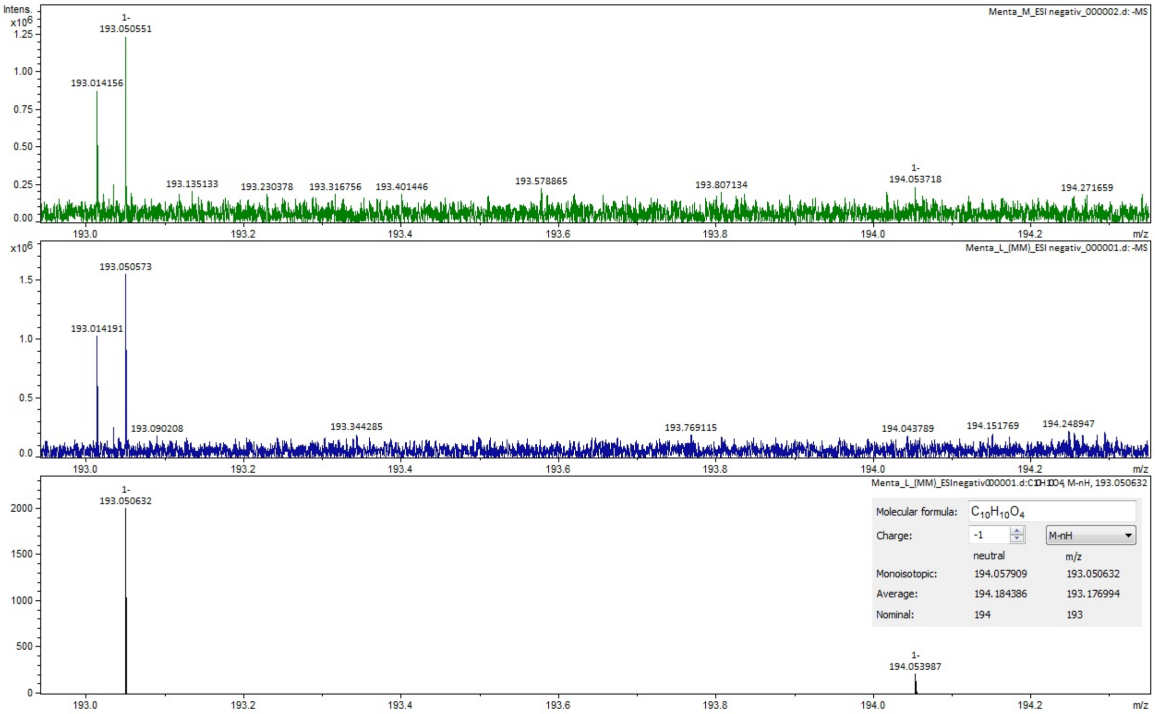

Figure S26. Ferulic acid ($C_{15}H_{10}O_6$) – m/z is 193.05, ESI-.

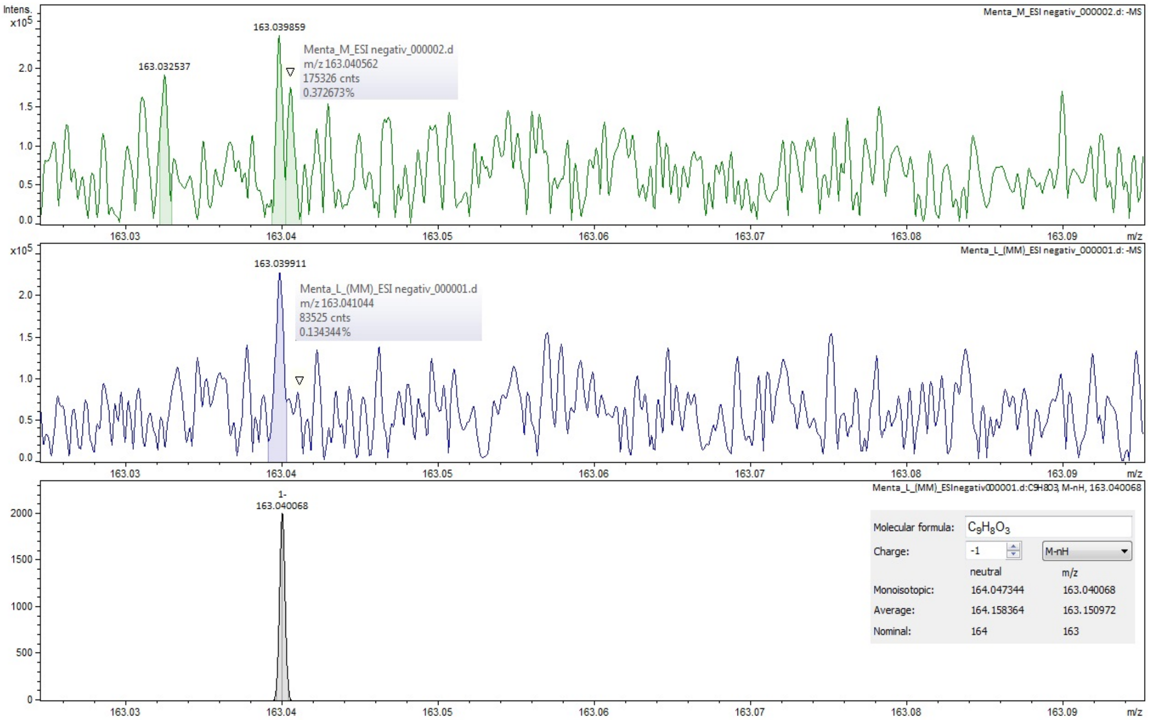

Figure S27. *p*-Coumaric acid ($C_9H_8O_3$) – m/z is 163.04, ESI-.

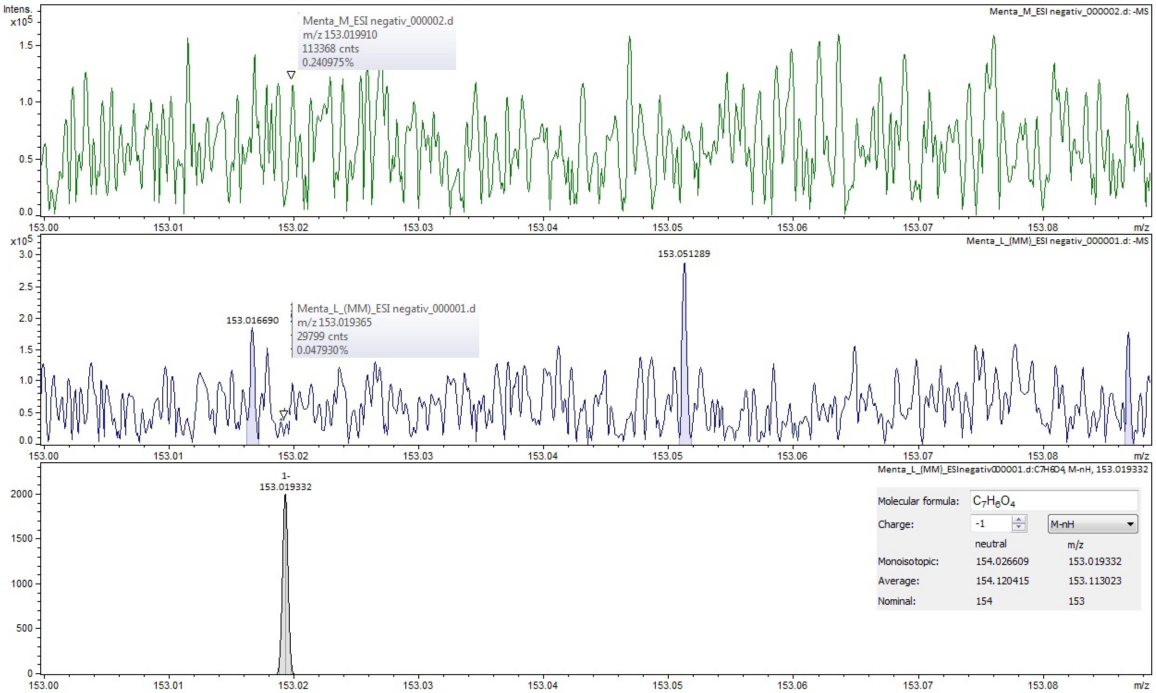

Figure S28. Protocatechuic acid ($C_7H_6O_4$) – m/z is 153.02, ESI-.

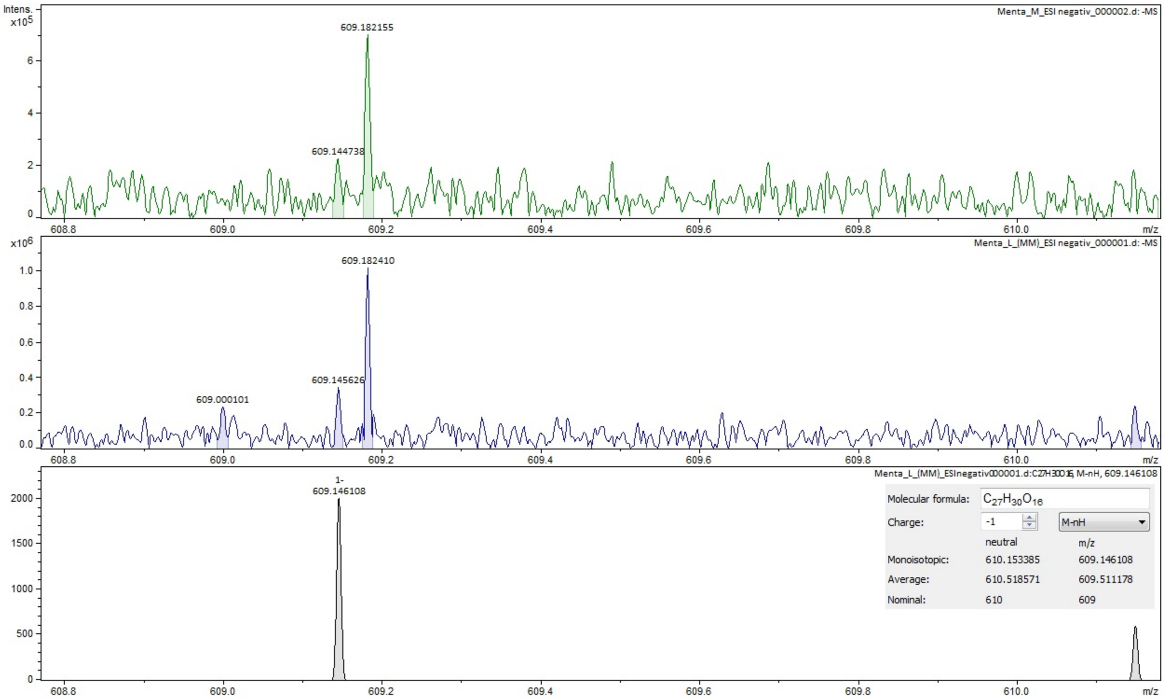

Figure S29. Rutin ($C_9H_8O_3$) – m/z is 609.15, ESI–.

Melissa officinalis L.

Green colour chromatogram – MLM E (lemon balm extract from control crop)

Brown colour chromatogram – MLF E (lemon balm extract from common crop)

Grey colour chromatogram – theoretical peak obtained by the computer program

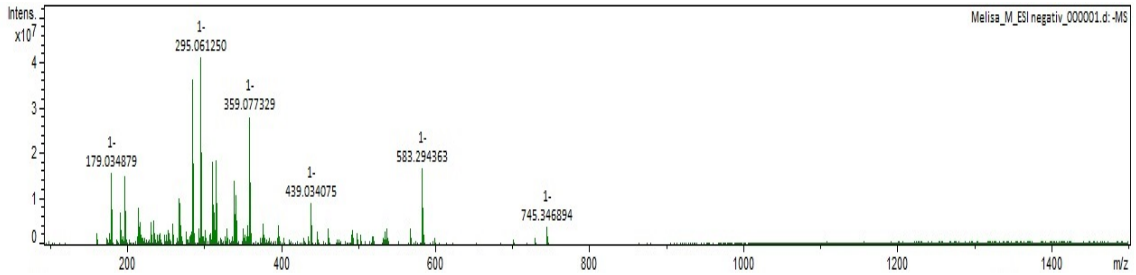

a. MLM E mass spectrum

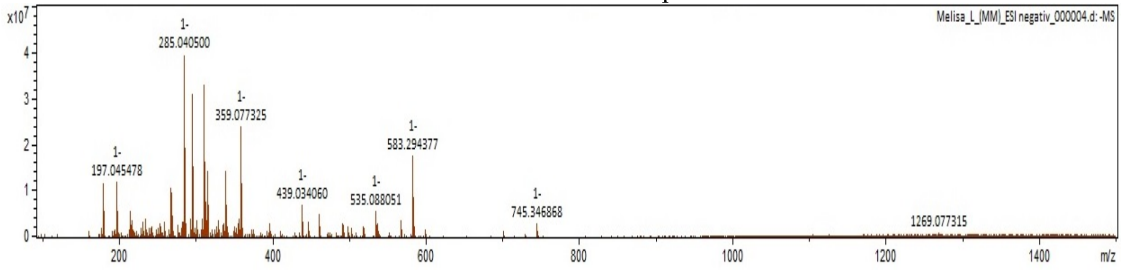

b. MLF E mass spectrum

Figure S30. (a) and (b) – entire mass spectra obtained on negative ionization.

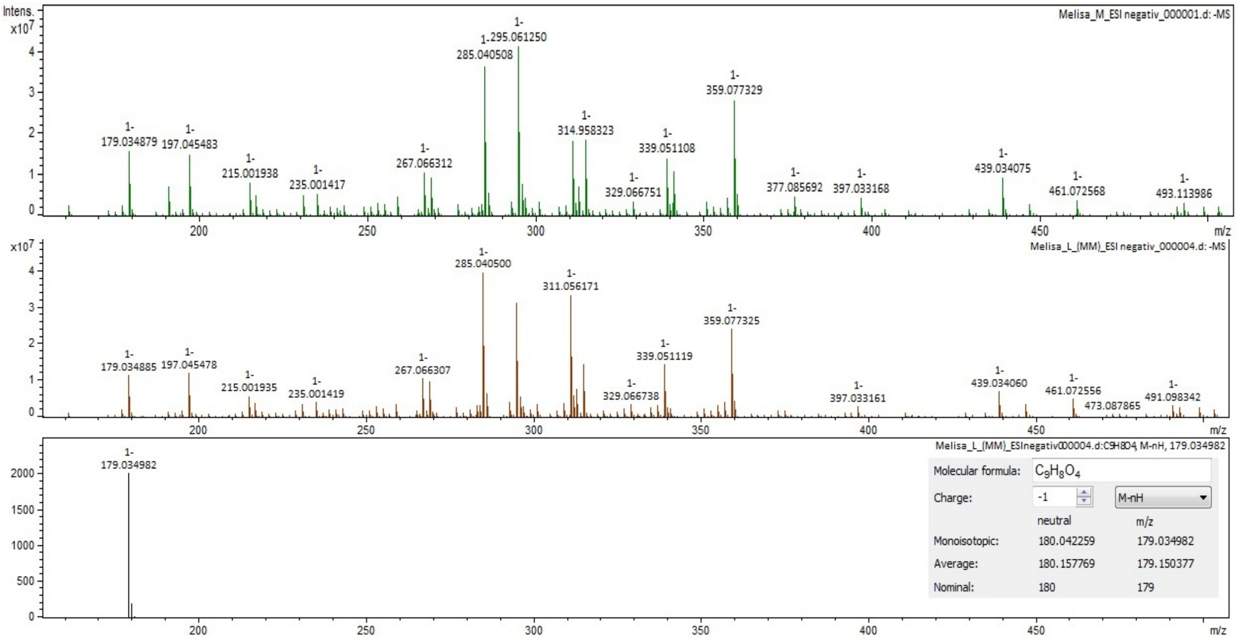

Figure S31. Caffeic acid ($C_9H_8O_4$) – entire chromatogram, m/z is 179.03, ESI-.

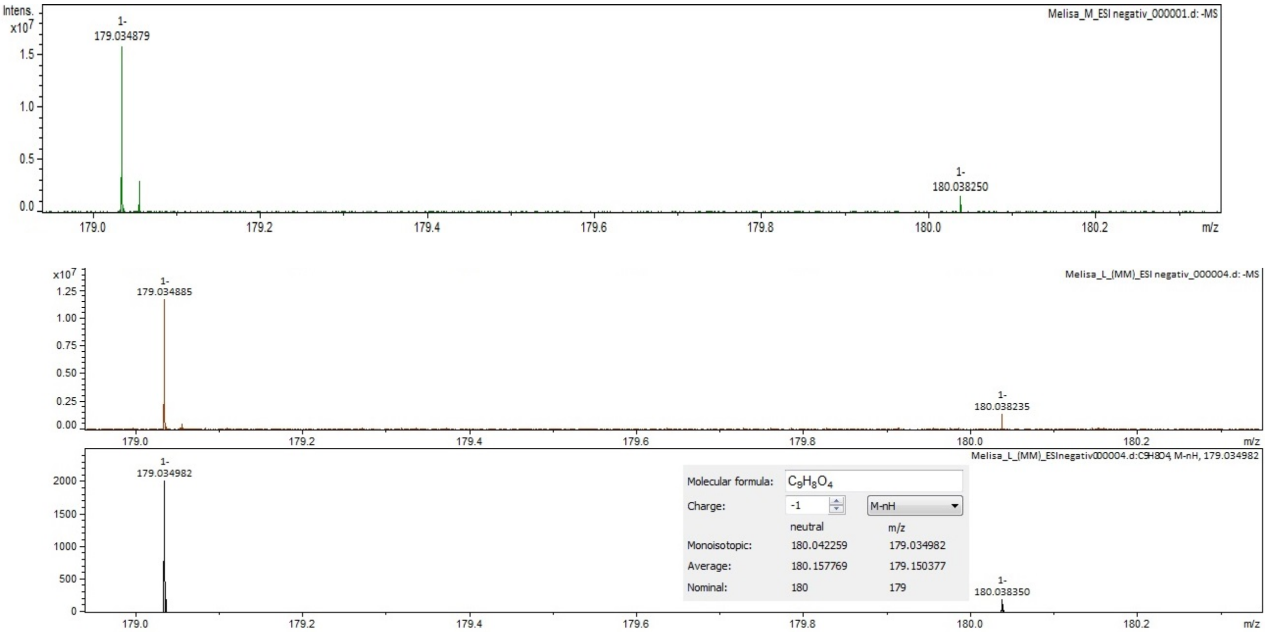

Figure S32. Caffeic acid ($C_9H_8O_4$) – m/z is 179.03, ESI -.

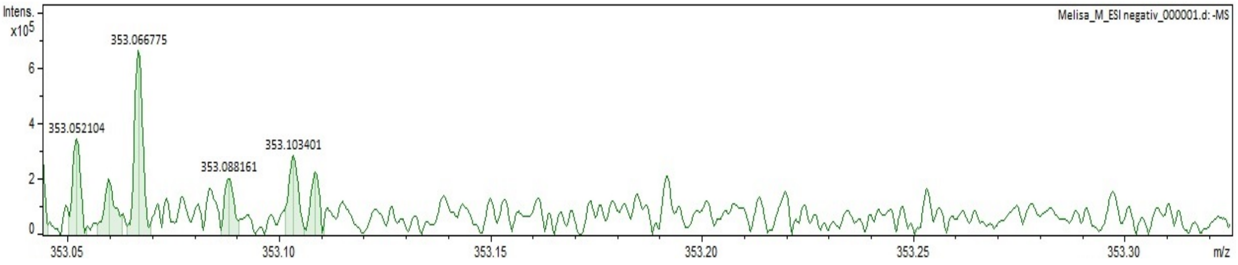

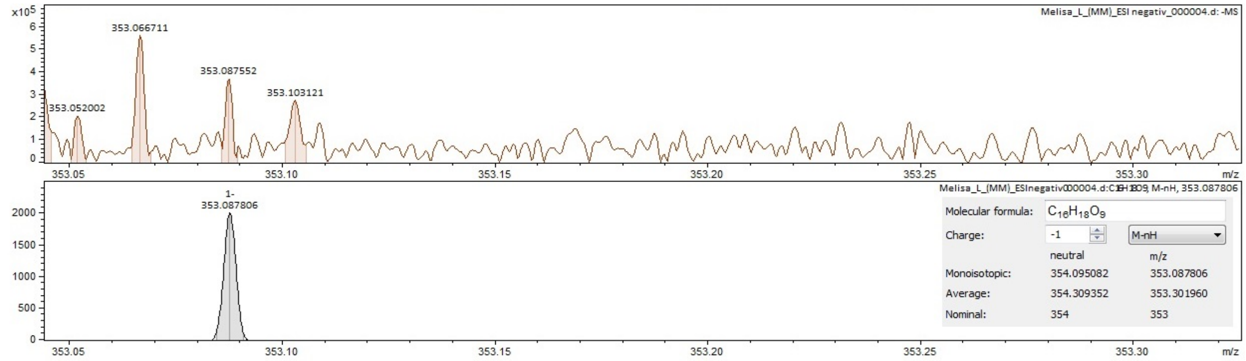

Figure S33. Chlorogenic acid ($C_{16}H_{18}O_9$) – m/z is 353.09, ESI –.

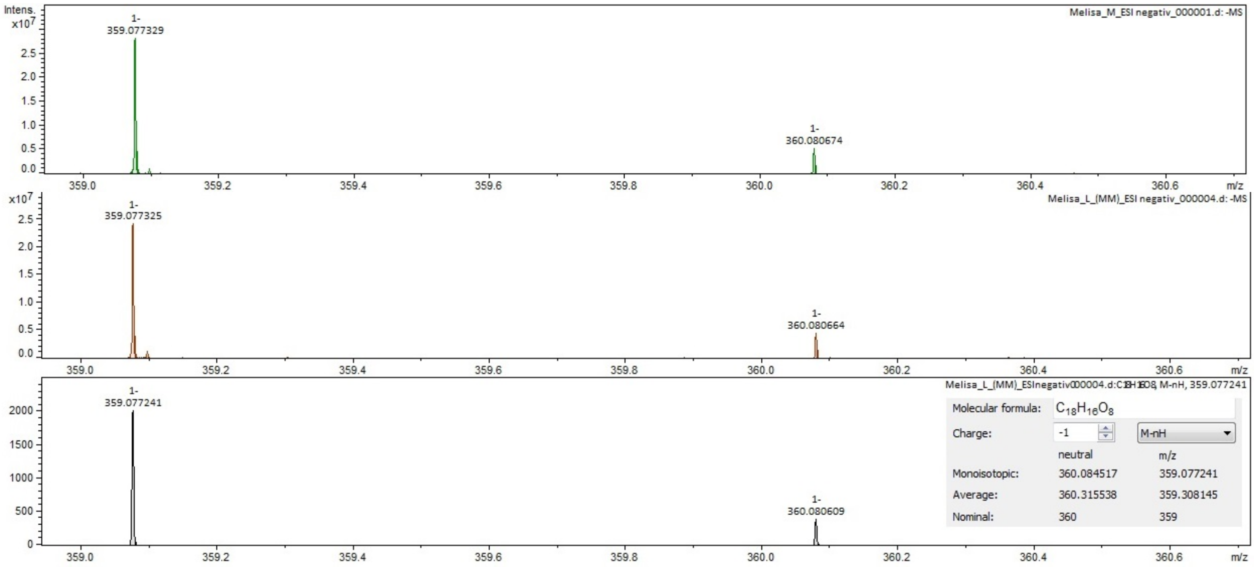

Figure S34. Rosmarinic acid ($C_{18}H_{16}O_8$) – m/z is 359.08, ESI-.

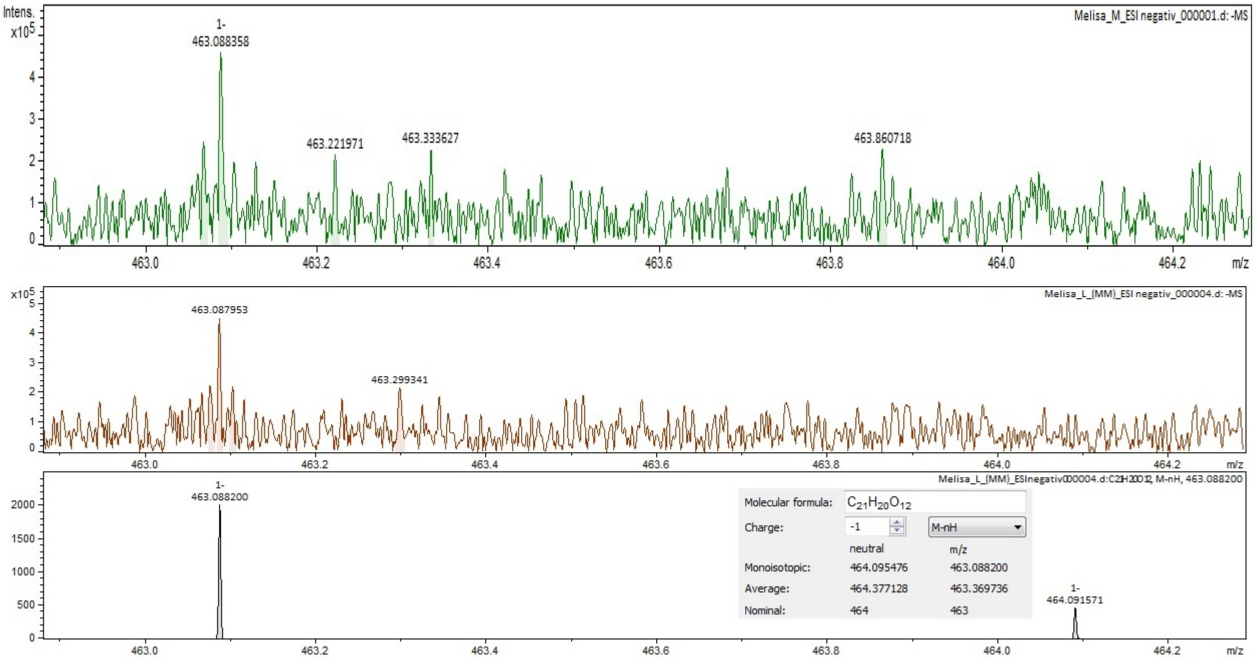

Figure S35. Isoquercitrin ($C_{21}H_{20}O_{12}$) – m/z is 463.09, ESI–.

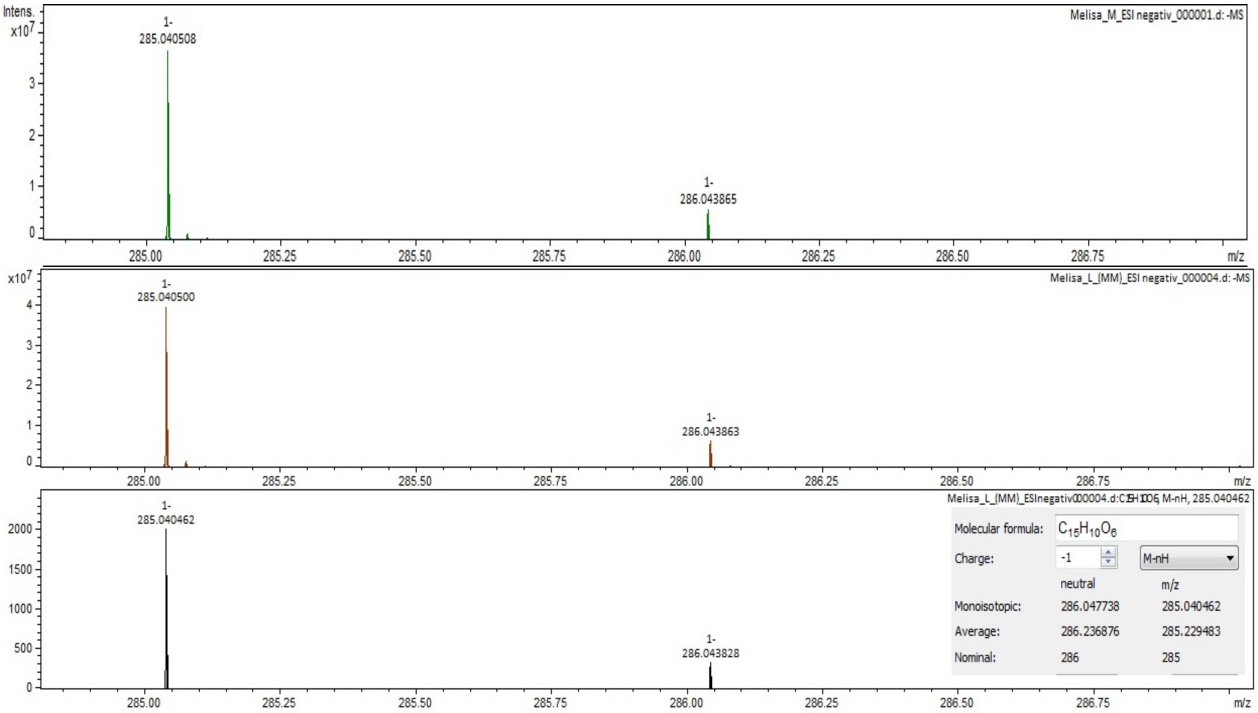

Figure S36. Luteolin + Kaempferol ($C_{15}H_{10}O_6$) – m/z is 285.04, ESI-.

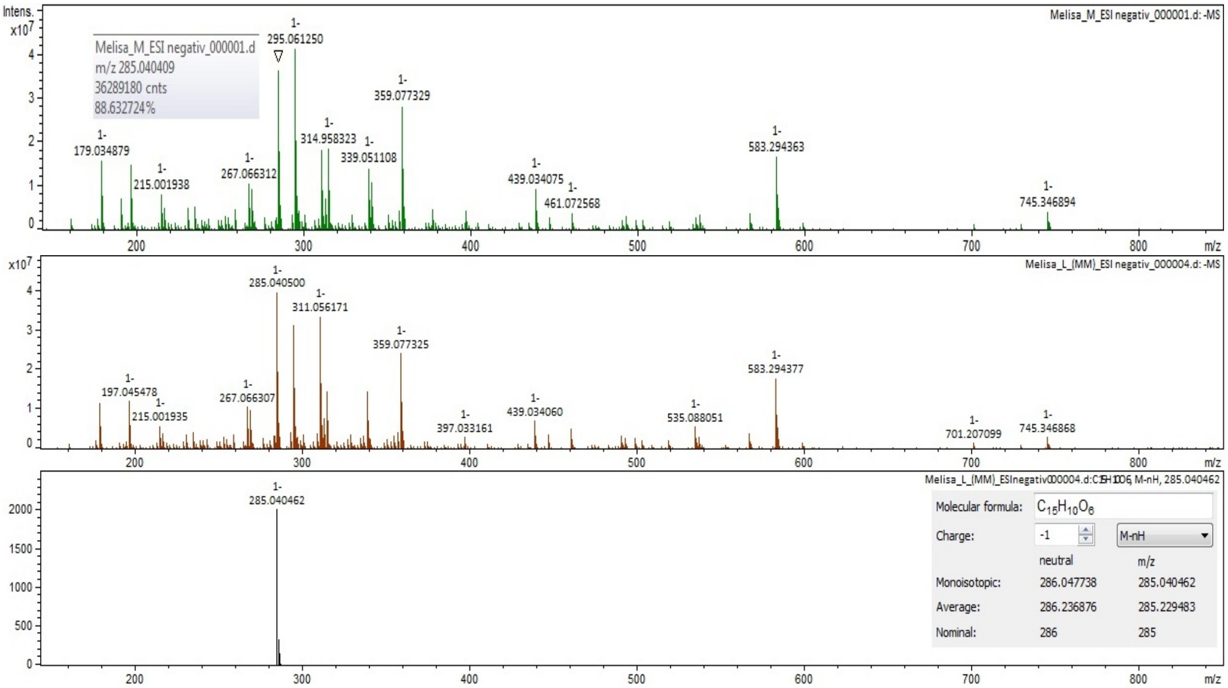

Figure S37. Luteolin + Kaempferol ($C_{15}H_{10}O_6$) – entire chromatogram, m/z is 285.04, ESI-.

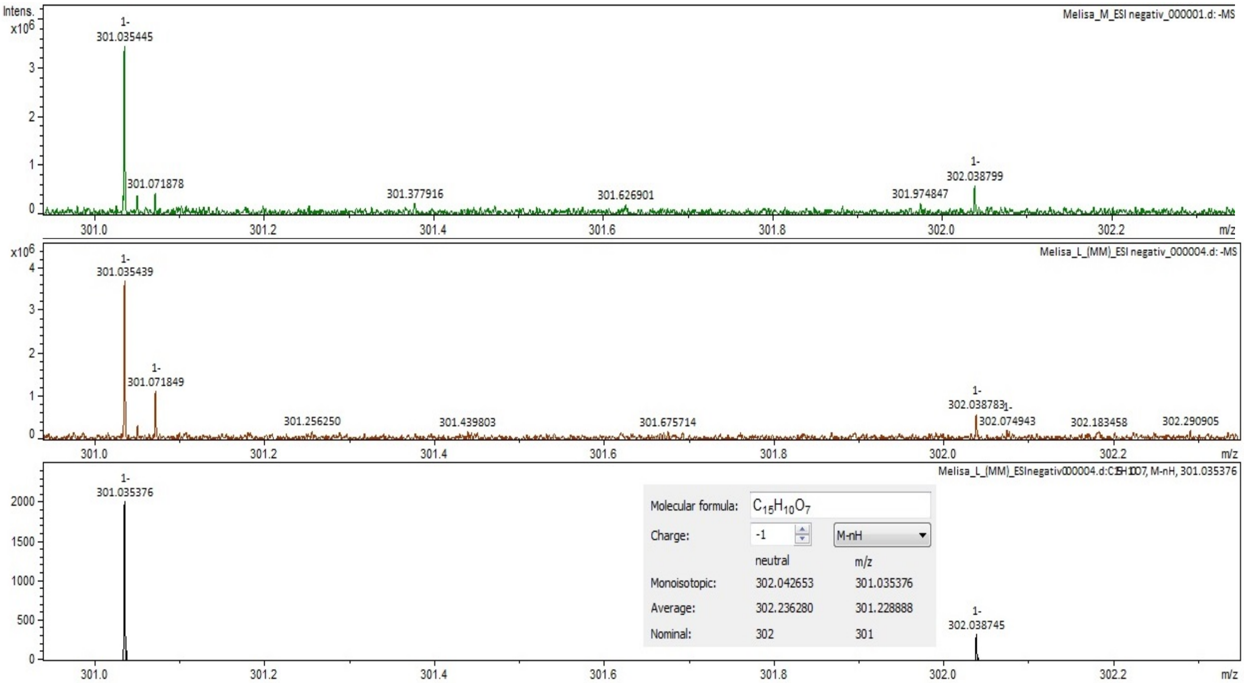

Figure S38. Quercetin ($C_{15}H_{10}O_7$) – m/z is 301.04, ESI-.

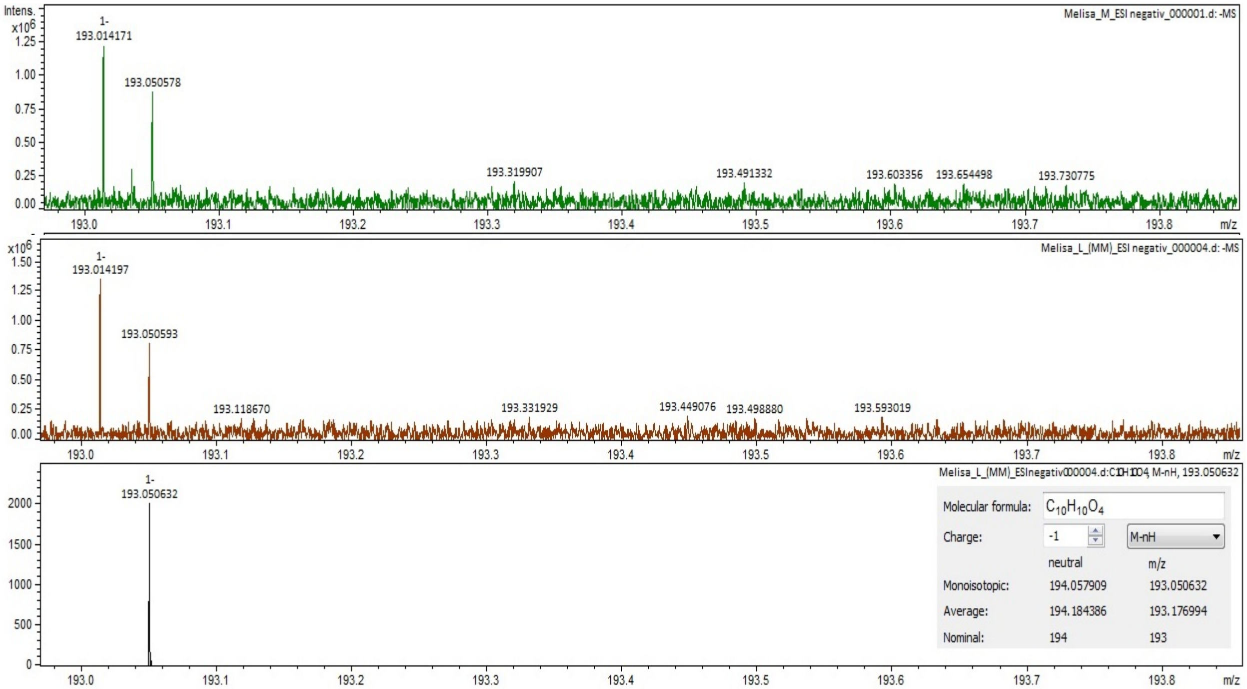

Figure S39. Ferulic acid ($C_{15}H_{10}O_6$) – m/z is 193.05, ESI-.

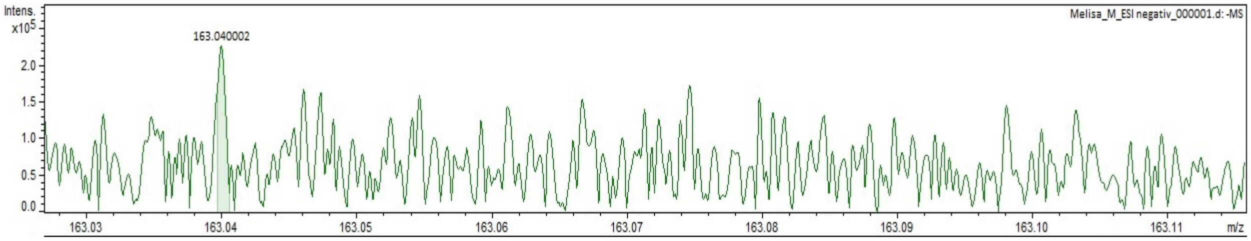

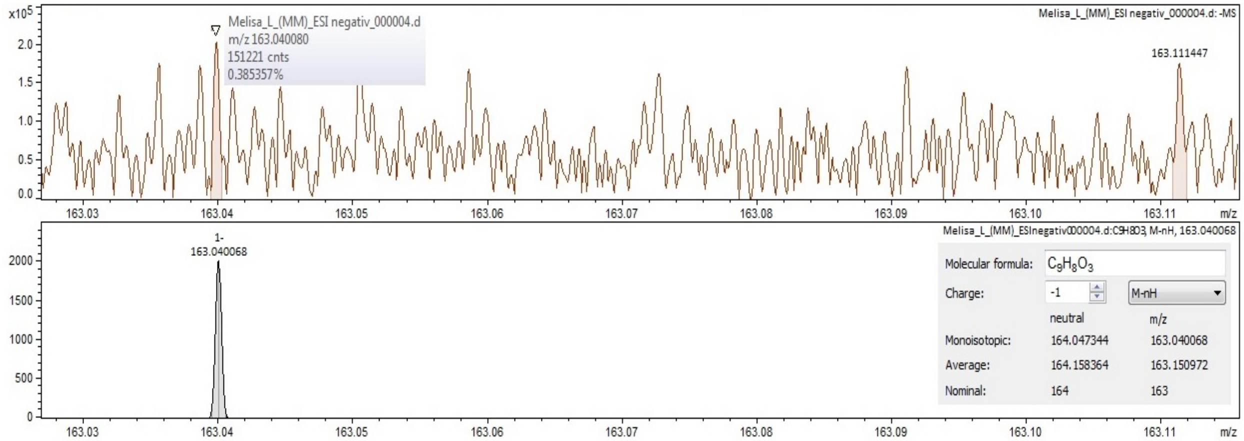

Figure S40. *p*-Coumaric acid ($C_9H_8O_3$) – m/z is 163.04, ESI-.

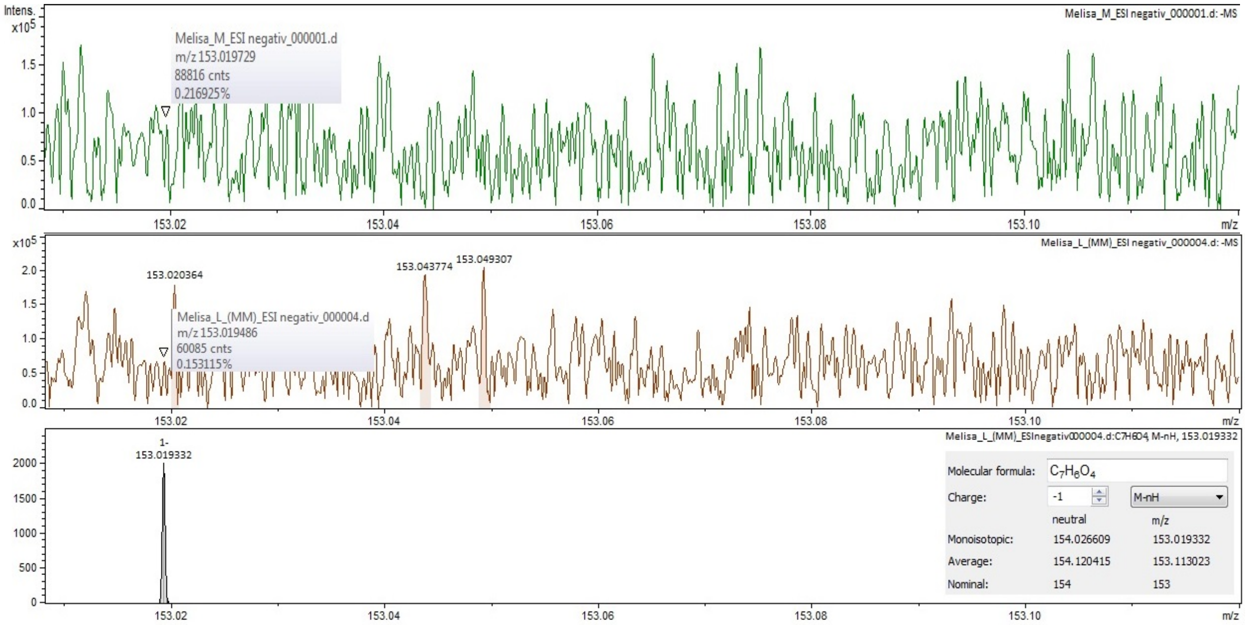

Figure S41. Protocatechuic acid ($C_7H_6O_4$) – m/z is 153.02, ESI-.

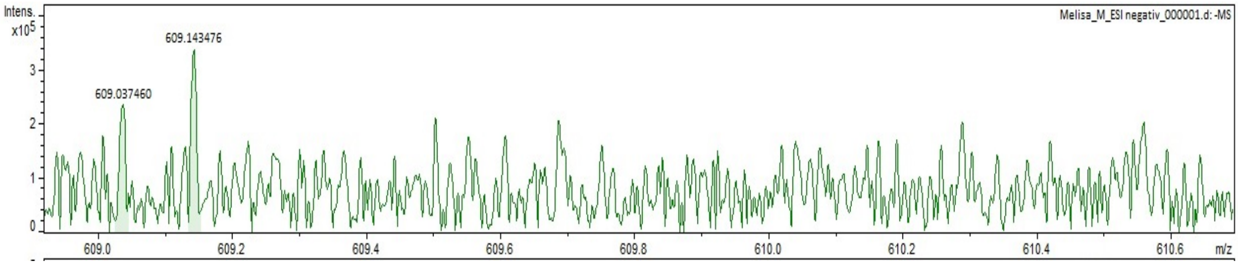

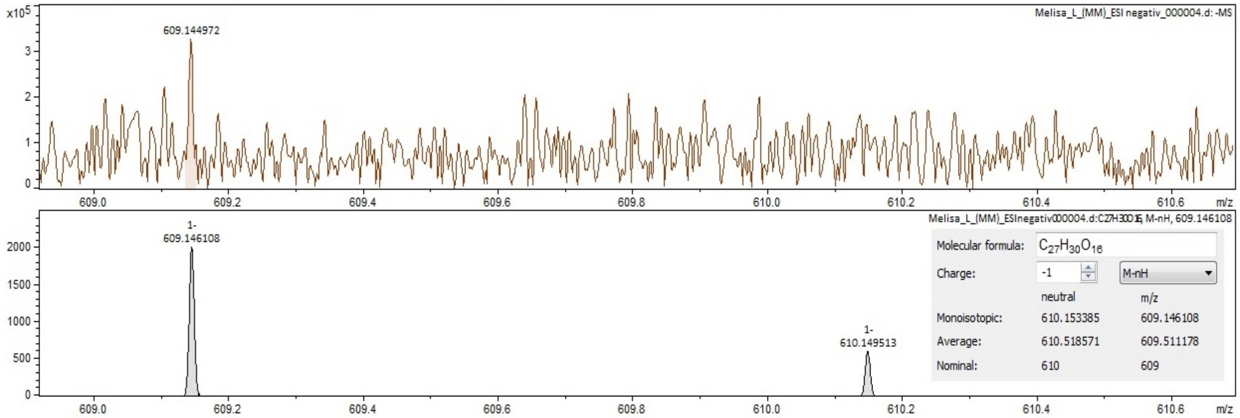

Figure S42. Rutin ($C_9H_8O_3$) – m/z is 609.15, ESI–.

S1.2. Statistical analysis

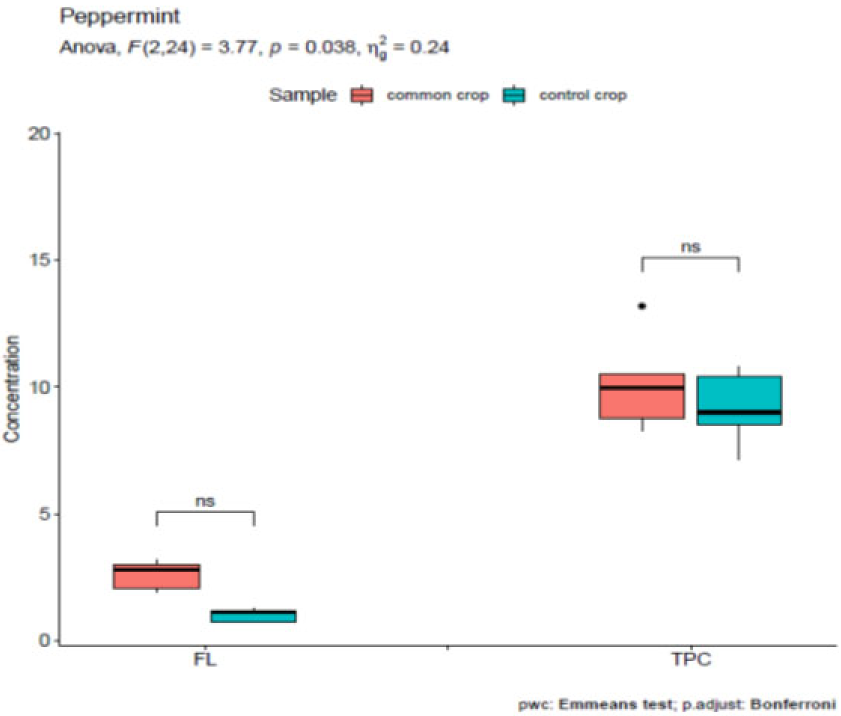

Figure S43. A two–way interaction boxplot for polyphenols assessed in peppermint.

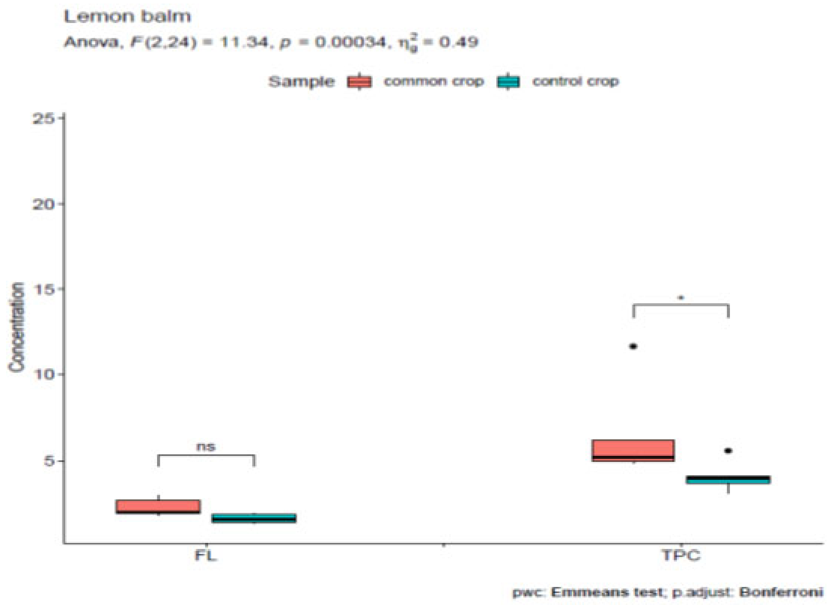

Figure S44. A two–way interaction boxplot for polyphenols assessed in lemon balm.

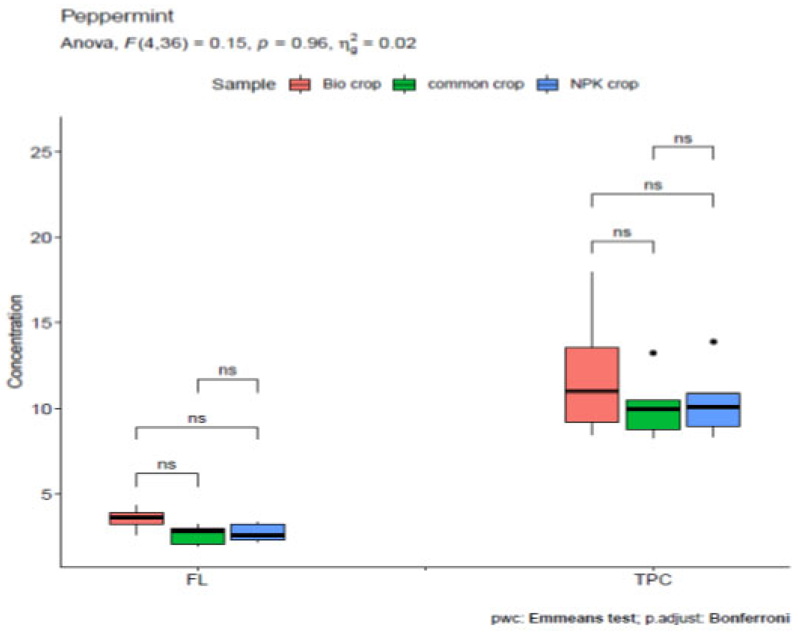

Figure S45. A two–way interaction boxplot for peppermint in relation to the type of fertilizer.

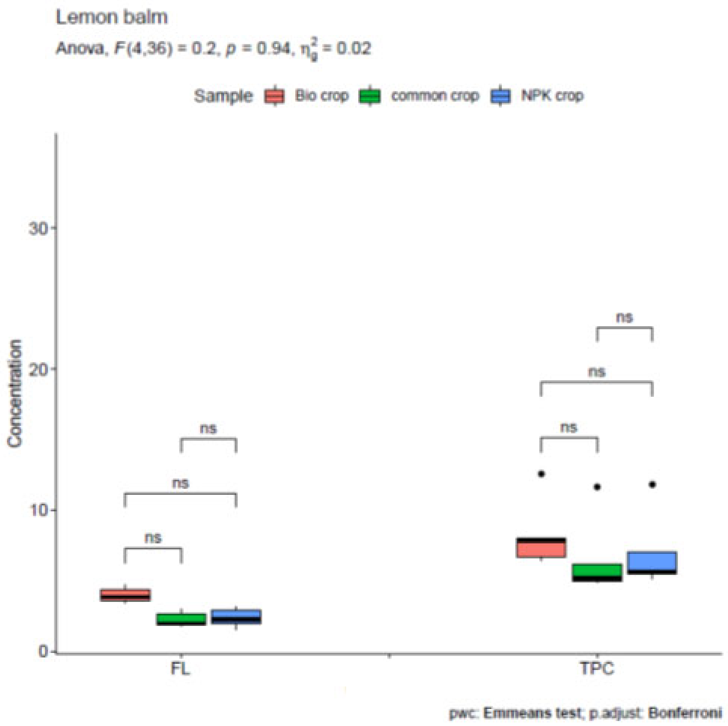

Figure S46. A two–way interaction boxplot for lemon balm in relation to the type of fertilizer.

S1.3. Ascorbic acid standard and correlation with the antioxidant activity

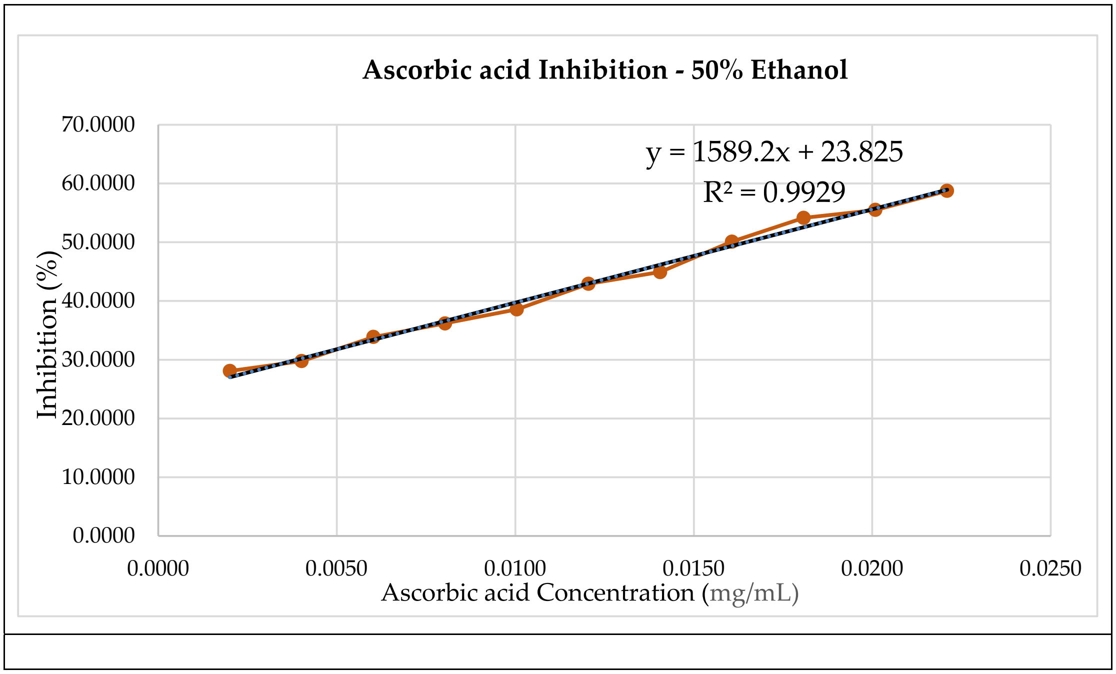

Figure S47. Calibration curve for ascorbic acid (vitamin C) - Antioxidant action in 50% ethanol.

S1.4. Trolox standard and correlation with the antioxidant activity

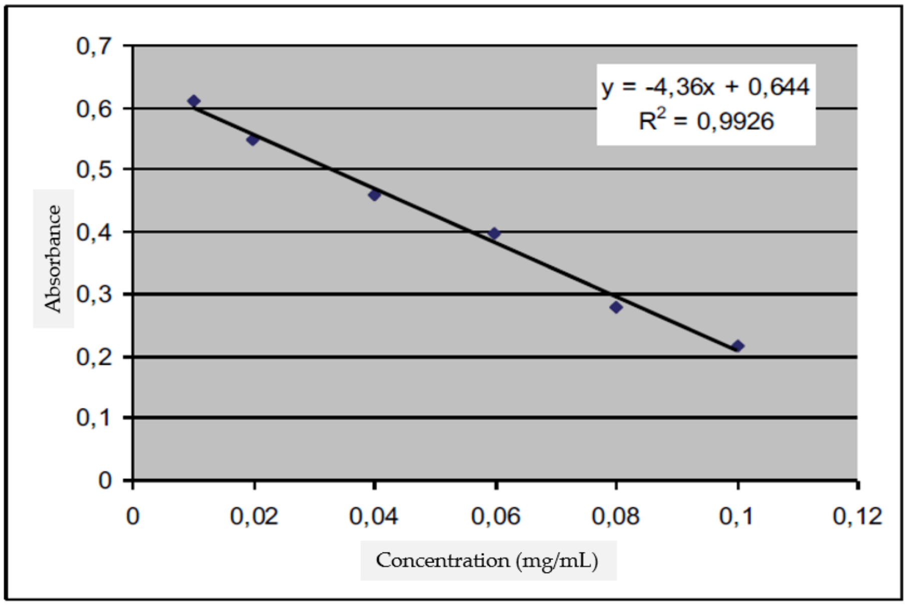

Figure S48. Calibration curve for trolox - Antioxidant action in 50% ethanol.

S1.5. Ferrous sulfate standard and correlation with the antioxidant activity

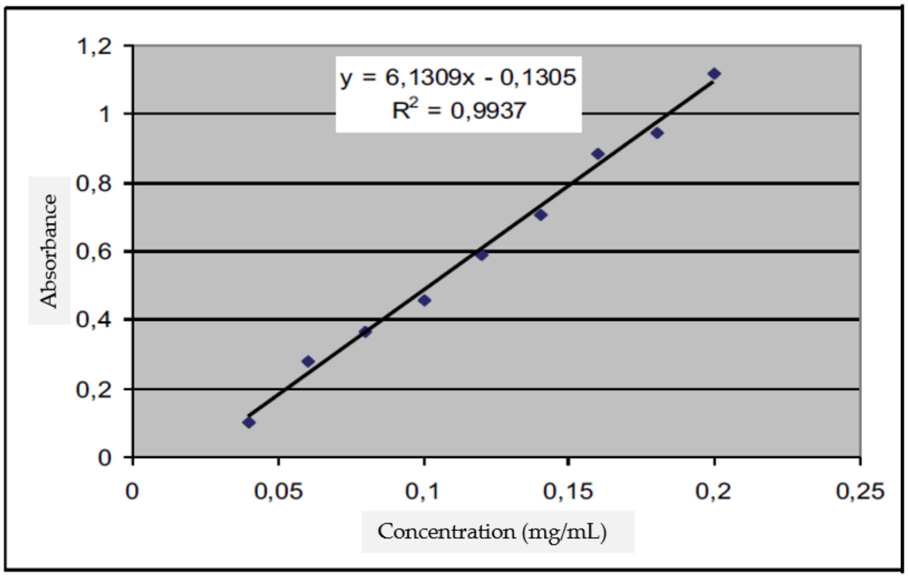

Figure S49. Calibration curve for ferrous sulfate - Antioxidant action in 50% ethanol.

Table S1. Correlation coefficients between TFL, TPC and antioxidant assay methodologies.

Correlation	r	R^2	R^2 (%)
**TFL *vs.* DPPH**	**-0,62**	0,39	38,81
**TFL *vs.* ABTS**	**-0,68**	0,46	46,10
**TFL *vs.* FRAP**	**-0,94**	0,87	87,42

**TPC *vs.* DPPH**	**-0,68**	0,46	46,38
**TPC *vs.* ABTS**	**-0,64**	0,41	41,22
**TPC *vs.* FRAP**	**-0,89**	0,80	79,92

DPPH: 2,2-diphenyl-1-picryl-hydrazine; **ABTS**: 2,20-azinobis-3-ethylbenzotiazoline-6-sulfonic acid; **FRAP**: ferric reducing antioxidant power; **r**: Pearson correlation coefficient; **R2**: coefficient of determination; **R2** (%): coefficient of determination expressed as a percentage.

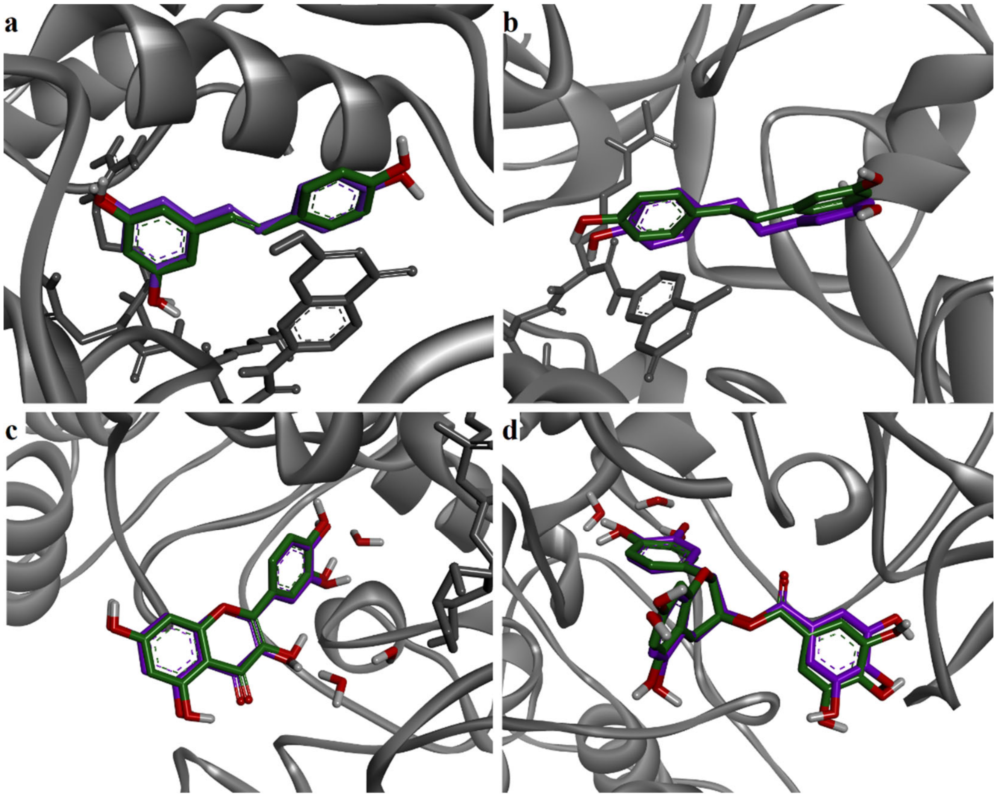

Figure S50. Superposition of predicted poses (purple) on initial conformations (green). (a) – SIRT1-resveratrol; (b) – SIRT5–resveratrol; (c) – SIRT6–quercetin; (d) – SIRT6–catechin gallate.

Table S2. Predicted dissociation constants (Kd) calculated using molecular docking experiments.

**Ligand**	**Kd (μM)**			
	**SIRT1 (activator)**	**SIRT5 (activator)**	**SIRT6 (activator)**	**SIRT6 (inhibitor)**
Caffeic acid	5.402	27.400	25.182	19.984
Chlorogenic acid	1.307	5.171	14.307	4.752
Ferulic acid	4.208	36.321	64.040	29.913
Isoquercitrin	0.255	0.968	18.397	50.222
Kaempferol	0.063	2.764	12.479	10.594
Luteolin	0.105	1.458	19.716	4.618
p-Coumaric acid	3.920	64.148	7.688	47.342
Protocatechuic acid	50.819	60.981	27.032	66.015
Quercetin	0.163	2.903	22.989*	8.579

Rosmarinic acid	0.086	0.749	1.999	0.842
Rutin	0.076	0.377	2.584	1.513
Resveratrol*	0.144	150.000	-	-
Catechin gallate*	-	-	-	0.398

* – positive control.

Table S3. Predicted free energies of binding using MM/PBSA calculations after 1 ns molecular dynamics simulations.

Ligand	Free energy of binding (kcal/mol)			
	SIRT1 (activator)	SIRT5 (activator)	SIRT6 (activator)	SIRT6 (inhibitor)
Rosmarinic acid	-30.038	-57.254	-62.288	-0.281
Resveratrol*	7.564	-45.598	-	-
Quercetin*	-	-	-56.360	-
Catechin gallate*	-	-	-	-4.543

* – positive control.

S.2. Materials and methods

S.2.1. Assessment of control soil composition

Soil samples have been taken from several areas at a depth of 10-15 cm and the concentrations of micro and macroelements were assessed. These determinations were done at Physico-Chemical Analysis Laboratory for Soil Sciences, Agrochemistry and Environmental Protection (LAFC) within the National Research-Development Institute for Pedology, Agrochemistry and Environmental Protection (ICPA) represented by Head of Laboratory, Dr. Nicoleta Vrînceanu, as executor of the tests.

The date of sample receiving was 06.17.2021, and the date of completion was 07.07.2021.

The parameters evaluated to determine soil quality were: determination of pH in aqueous suspension 1: 25 (STAS 7184–13), humus content by wet oxidation (STAS 7184/21–82; PTL 12), total nitrogen (Kjeldahl method; STAS 7184/2–85), mobile phosphorus (P – ammonium acetate–lactate extraction; STAS 7184/19–82; PTL 19), potassium (K – ammonium acetate–lactate extraction; STAS 7184/18–80; PTL 22), determination of electrical conductivity and estimation of total soluble salt content (Res. cond. – STAS 184/7–87 chap. 3.2; PTL 07), and the content of mobile forms (copper, iron, manganese, zinc) extractable in EDTA solution (PTL 32, ed.2 and 3, rev. 0 and 1).

S.2.2. Assessment of fertilized soil composition

Formula of Bio–Fertil, 20 (produced in Romania by controlled decomposition of manure): nitrogen (N) – 30 mg/100 g; potassium (K) – 5.4 mg/100 g; phosphorus (P) – 6 mg/100 g; zinc (Zn) – 102 mg/Kg; copper (Cu) – 4.6 mg/Kg; iron (Fe) – 10.1 mg/Kg; pH –7; organic matter – minimum 50%.

The NPK 20–20–20 fertilizer mixture contains the following trace elements: 20% Nitrogen (N) (Nitric nitrogen – 5.5%, Ammonium nitrogen – 2.5%, Urea nitrogen – 12.0%), Phosphoric anhydride (P_2O_5) soluble in water (20%), Phosphoric anhydride (P_2O_5) soluble in neutral ammonium citrate and water (20%), Potassium oxide (K_2O) soluble in water (20%), Boron (B) soluble in water (0.020%), Copper (Cu) soluble in water (0.004%) [100% chelated with EDTA – 0.004%], Iron (Fe) soluble in water (0.041%) [66% chelated with EDTA – 0.027%, 34% chelated with EDDHA – 0.014%], Manganese (Mn) soluble in water (0.025%) [100% EDTA chelated – 0.025%], Molybdenum (Mo) soluble in water (0.002%), Zinc (Zn) soluble in water (0.015%) [100% EDTA chelated – 0.015%].

S.2.3. Assessment of microelements from fertilized crops

The date of sample receiving was 07.12.2021, and the date of completion was 08.05.2021.

Nitrogen (Kjeldahl method; SR EN ISO 20483:2014; PTL 11), phosphorus (colorimetric method, with ammonium metavanadate, PTL 20), potassium and calcium (flame spectroscopic determination, PTL 24), magnesium (flame atomization atomic absorption spectrometry, PTL 24), copper, iron, manganese, zinc (atomic absorption spectrometry PTL 30) were determined.

S.2.4. UHPLC methodology

The method used to prepare injection solutions was as follows: 1 mg/mL stock solutions of each standard compound were obtained in methanol and kept refrigerated at 4°C until use. The concentration of each level of the calibration curves are presented in Table S4. The solutions were obtained by diluting in the first line of the mobile phase gradient (methanol-water, 9:1). The samples were dissolved in the same mixture of methanol-water (9:1) and filtered through 0.2 µm syringe filters (Acrodisc MS Syringe Filters WWPTFE Membrane) prior to injection..

Table S4. Calibration curve concentration by level (expressed in µg/g) and purity (%).

**Level**	**PRO 99%**	**CHL 98%**	**CAF 99%**	**COU 99%**	**FER 98%**	**RUT 94%**	**ISO 87%**	**ROS 96%**	**LUT 99%**	**QUE 95%**	**KAE 99%**
1	5.05	4.90	4.95	5.00	4.90	4.70	2.18	4.80	1.98	4.75	0.20
2	10.10	9.80	9.90	10.00	9.80	9.40	4.35	9.60	3.96	9.50	0.40
3	20.20	19.60	19.80	20.00	19.60	18.80	8.70	19.20	7.92	19.00	0.79
4	50.49	49.00	49.50	50.00	49.00	47.00	21.75	48.00	19.80	47.50	1.98

PRO – Protocatechuic acid, RUT – Rutin, CAF – Caffeic acid, CHL – Chlorogenic acid, LUT – Luteolin, KAE – Kaempferol, ROS – Rosmarinic acid, QUE – Quercetin, ISO – Isoquercitrin, FER – Ferulic acid, COU – *p*-Coumaric acid.

LC conditions

All separations were performed on the ACQUITY Arc System equipped with an ACQUITY QDa Detector. Empower 3 Software was used for data acquisition and processing. The Column used is a CORTECS C_{18}, 4.6×50 mm, 2.7 µm. Solvents used were ammonium formate 10 mmol (solvent A) and acetonitrile (solvent B) with a flow rate of 0.5 mL/min. The volume injected was 5 µL and analysis time was 21 min. The gradient conditions were: 0 min 8% B, 8 min 20% B, 16 min 27% B, 19 min 60% B, 20 min 60% B, 21 min 8% B.

MS conditions

Ionization mode ESI-

All compounds were satisfactorily separated. Chromatogram and retention times are presented below (Figure S51 and Table S5).

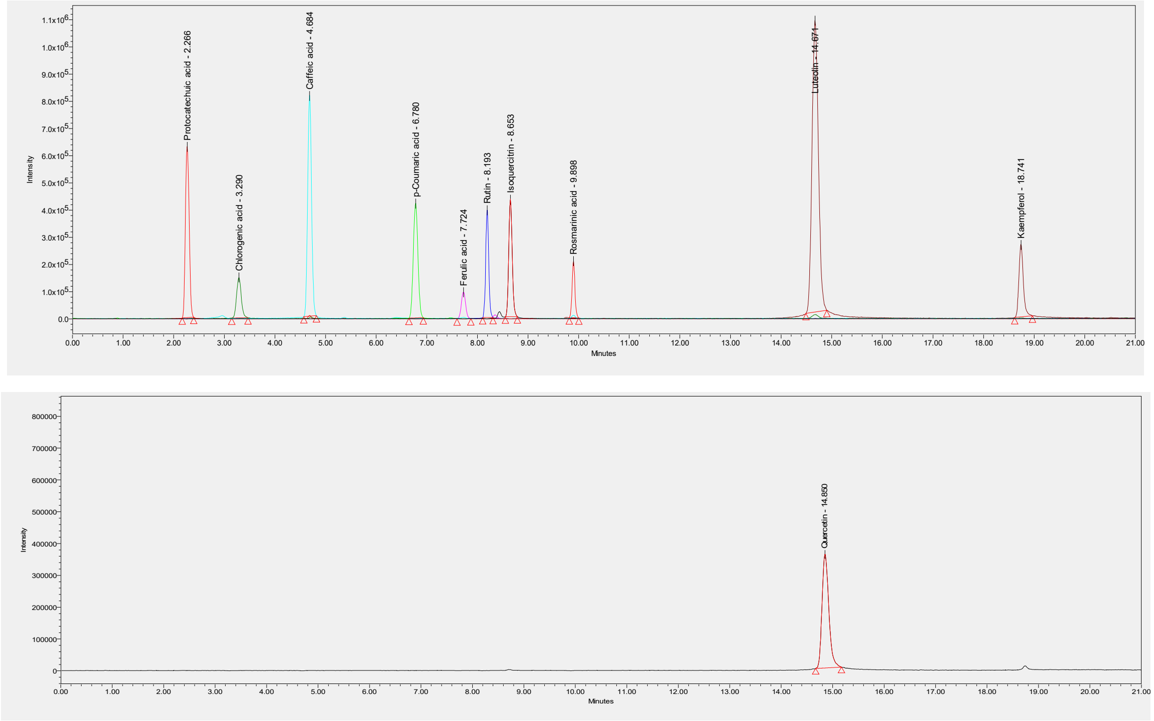

Figure S51. Standard chromatogram.

Table S5. Retention times.

Compound name	Retention time (Rt-Min)
Protocatechuic acid	2.266
Chlorogenic acid	3.290
Caffeic acid	4.684
p-Coumaric acid	6.780
Ferulic acid	7.724
Rutin	8.193
Isoquercitrin	8.653
Rosmarinic acid	9.898
Luteolin	14.671
Quercetin	14.850
Kaempferol	18.741
